# Effects of High-Intensity Interval Exercise versus Moderate Continuous Exercise on Glucose Homeostasis and Hormone Response in Patients with Type 1 Diabetes Mellitus Using Novel Ultra-Long-Acting Insulin

**DOI:** 10.1371/journal.pone.0136489

**Published:** 2015-08-28

**Authors:** Othmar Moser, Gerhard Tschakert, Alexander Mueller, Werner Groeschl, Thomas R. Pieber, Barbara Obermayer-Pietsch, Gerd Koehler, Peter Hofmann

**Affiliations:** 1 Department of Internal Medicine, Division of Endocrinology & Metabolism, Medical University of Graz, Graz, Austria; 2 Institute of Sports Sciences, Exercise Physiology & Training Research Group, University of Graz, Graz, Austria; 3 Center of Sports Medicine & Sports Orthopedics, University Outpatient Clinic, University of Potsdam, Potsdam, Germany; University of Milan, ITALY

## Abstract

**Introduction:**

We investigated blood glucose (BG) and hormone response to aerobic high-intensity interval exercise (HIIE) and moderate continuous exercise (CON) matched for mean load and duration in type 1 diabetes mellitus (T1DM).

**Material and Methods:**

Seven trained male subjects with T1DM performed a maximal incremental exercise test and HIIE and CON at 3 different mean intensities below (A) and above (B) the first lactate turn point and below the second lactate turn point (C) on a cycle ergometer. Subjects were adjusted to ultra-long-acting insulin Degludec (Tresiba/ Novo Nordisk, Denmark). Before exercise, standardized meals were administered, and short-acting insulin dose was reduced by 25% (A), 50% (B), and 75% (C) dependent on mean exercise intensity. During exercise, BG, adrenaline, noradrenaline, dopamine, cortisol, glucagon, and insulin-like growth factor-1, blood lactate, heart rate, and gas exchange variables were measured. For 24 h after exercise, interstitial glucose was measured by continuous glucose monitoring system.

**Results:**

BG decrease during HIIE was significantly smaller for B (p = 0.024) and tended to be smaller for A and C compared to CON. No differences were found for post-exercise interstitial glucose, acute hormone response, and carbohydrate utilization between HIIE and CON for A, B, and C. In HIIE, blood lactate for A (p = 0.006) and B (p = 0.004) and respiratory exchange ratio for A (p = 0.003) and B (p = 0.003) were significantly higher compared to CON but not for C.

**Conclusion:**

Hypoglycemia did not occur during or after HIIE and CON when using ultra-long-acting insulin and applying our methodological approach for exercise prescription. HIIE led to a smaller BG decrease compared to CON, although both exercises modes were matched for mean load and duration, even despite markedly higher peak workloads applied in HIIE. Therefore, HIIE and CON could be safely performed in T1DM.

**Trial Registration:**

ClinicalTrials.gov NCT02075567 http://www.clinicaltrials.gov/ct2/show/NCT02075567

## Introduction

In support of a regular insulin therapy, physical activity and exercise training have shown to positively affect patients with type 1 diabetes mellitus (T1DM) reducing the risk of all-cause mortality and cardiovascular disease [1] and cancer [2, 3]. However, T1DM patients often refuse to perform exercise due to the increased probability of experiencing a hypoglycemic event. In order to avoid hypoglycemia during and after exercise, the dose of both basal long-acting insulin and short-acting insulin, as well as the choice of exercise intensity and duration, is of high relevance and must be balanced. Rabasa-Lhoret et al. [4] showed that for continuous exercise at different intensities (25, 50, and 75% of maximal oxygen uptake (VO_2max_)), the risk of exercise-induced hypoglycemia could be minimized by an exercise intensity-dependent reduction of the short-acting premeal insulin administration (25, 50, and 100% of usual insulin dose) in subjects with T1DM.

It was recently shown that aerobic high-intensity interval exercise (HIIE) was equally or even more effective in improving oxidative capacity compared to conventional moderate continuous exercise (CON) in healthy subjects [5–7] and in patients suffering from chronic diseases [8–12]. These effects are suggested to be caused by markedly higher peak workloads during the intervals. Therefore, aerobic HIIE has become more focus of attention as an alternative training intervention strategy to standard CON even in rehabilitation programs. However, the methods to prescribe intermittent exercise individually, accurately, and safely are still under discussion. Tschakert et al. [13] and Tschakert and Hofmann [14] showed that the prescription of the single HIIE components, particularly the peak workload (P_peak_), the peak workload duration (t_peak_), and the mean load (P_mean_), determines the acute metabolic and cardiorespiratory response during exercise, which may represent potential health risks in patients [15]. Consequently, also the acute hormone response, blood glucose homeostasis during and after exercise, and corresponding health risks in subjects with T1DM may be strongly influenced by the HIIE protocol.

Patients with T1DM often avoid strenuous exercise for fear of hypoglycemia although several studies revealed an equal [16], or even a smaller, decline of BG concentration [17–20] yielded by exercise with at least one, or more, high-intensity work bouts compared to moderate continuous exercise.

However, these studies had considerable methodological limitations which we tried to overcome in our study design. First, HIIE and CON were not matched for mean load or total energy expenditure (except Iscoe and Riddell [16]) which didn’t allow a comparison of the acute responses between HIIE vs. CON.

Second, exercise intensity was prescribed by means of fixed percentages of VO_2max_ or maximal heart rate (HR_max_) (except for Iscoe and Riddell [16]) yielding heterogeneous exercise stimuli across subjects not considering individual metabolic conditions such as determined by turn points for blood lactate concentration or gas exchange data [21–24]. Individual and objective submaximal and maximal markers of aerobic performance such as the first (LTP_1_) and second lactate turn point (LTP_2_) and the maximal power output (P_max_) assessed in an incremental exercise test are recommended for prescribing exercise intensity for continuous and intermittent exercise [24]. This turn point concept [25] is embedded in the three phase concept of metabolism [26] and theoretically based on the lactate shuttle theory by Brooks [27].

In addition, with respect to short-acting insulin dose reduction strategies applied in several studies [4, 16, 18, 20], the amount of insulin reduction (no reduction or fixed reduction independent of exercise intensity) was inappropriate. Furthermore, the time span between the insulin administration and exercise (a time span that led to the full impact of short-acting insulin) was unsuitable to investigate the exercise-induced BG decrease and the risk of hypoglycemia yielded by different exercise modes.

In contrast, our study included a novel methodological approach as aerobic HIIE with short peak workload durations (short HIIE) and CON were matched for P_mean_ and total duration, and performed at three different mean intensities relative to the individual markers LTP_1_ and LTP_2_.

The main goal of this study was to investigate the homeostasis of BG and interstitial glucose, and the associated risk of hypoglycemia during and after aerobic HIIE compared to CON in trained subjects with T1DM. For this purpose, short-acting pre- and post-exercise insulin was reduced dependent on mean exercise intensity (P_mean_), and a new basal ultra-long-acting insulin was administered. A second goal of the study was to compare the acute response for BG homeostasis-relevant hormones such as adrenaline, noradrenaline, dopamine, cortisol, glucagon, and insulin-like growth factor-1, as well as for lactate (La), heart rate (HR), and gas exchange variables yielded by both exercise protocols.

We hypothesized no significant difference in BG decrease or risk of hypoglycemia during and after aerobic short HIIE and CON. In addition, we hypothesized that the acute response for hormones and other physiological parameters will not be significantly different between both exercise modes, despite significantly higher peak workloads in HIIE.

## Material and Methods

### Subject characteristics

**Fig 1:**
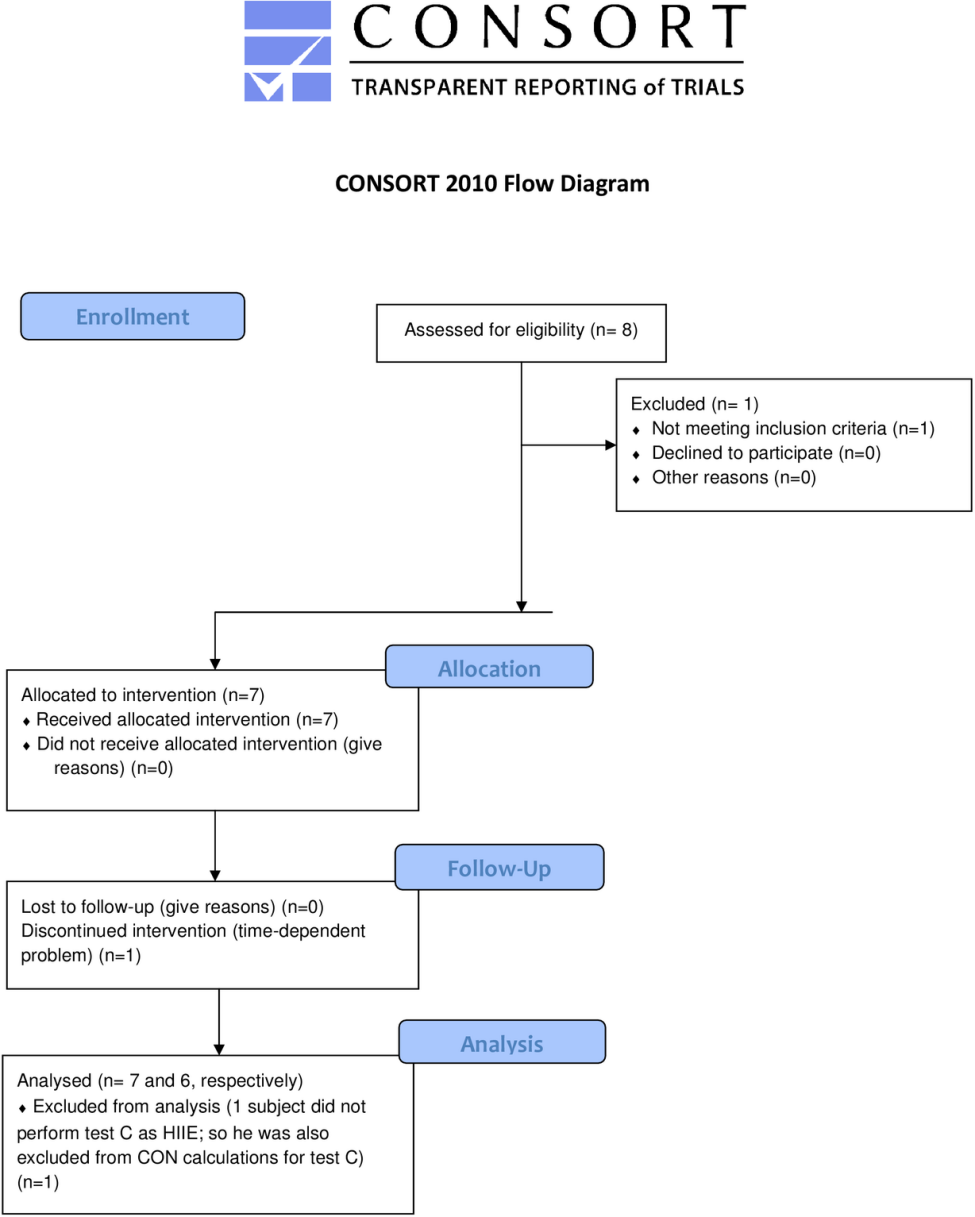
Consort flow diagram. CON: moderate continuous exercise, HIIE: short high-intensity interval exercise.

A total of seven eligible men with T1DM were enrolled in this trial (Fig 1). Participants’ anthropometric and performance characteristics, as well as diabetes-specific data, are shown in Tables 1 and 2. Subjects were trained (VO_2max_ = 52 ± 8.2 ml.kg^-1^.min ^-1^) and had no ECG or blood pressure abnormalities.

**Table 1:** Subject characteristics: BMI: body mass index. Values are given as mean and SD.

Age (years)	Height (m)	Weight (kg)	BMI (kg.m^-2^)
24 ± 5.3	1.76 ± 0.40	74 ± 5.1	23.9 ± 2.5

**Table 2:** Diabetes specific characteristics: HbA_1c_: hemoglobin A_1c_; c-peptide: connecting peptide; CarbF: carbohydrate factor; TDD: total daily dose of insulin. Values are given as mean and SD.

HbA_1c_ (% (mmol.mol^-1^))	c-peptide (nmol.l^-1^)	CarbF (g)	TDD (U)
7.4 (57) ± 0.6 (6.3)	0.13 ± 0.19	12 ± 4.9	41 ± 16

### Consent procedures

All participants gave their written informed consent before participating in any trial activities. The study was performed according to Good Clinical Practice and the Declaration of Helsinki (ClinicalTrials.gov ID: NCT02075567). The Ethics Committee of the Medical University of Graz approved the study design (registry number 26–069 ex 13/14). Measurements were permitted until January 17, 2015.

### Eligibility criteria and assessment

To be eligible for the study, participants were assessed for following criteria as shown in Table 3:

**Table 3:** (Testing day) inclusion and exclusion criteria.

Inclusion criteria		Exclusion criteria	
Over all	Testing day	Over all	Testing day
Male T1DM	No hypoglycemia 48h pre-exercise	Illness or disease, that confound results	Illness on or before the testing day
Diabetes duration > 12 months	No alcohol 24 h before testing	Use of drugs	BG before testing < 4.4 mmol.l^-1^
Aged: 18–35 years		Interference with insulin action	Technical difficulties with CGM
HbA_1c_ < 8% (< 64mmol.mol^-1^)		Addiction to alcohol	Incorrect time of bolus insulin injection
Fasting c-peptide < 0.3 nmol.l^-1^		Suspected allergy to trial products	
Intensified insulin or insulin pump		Mental incapacity	
No diabetic long-term complications		Other physical and/or mental disease	

Inclusion and exclusion criteria were documented in a standardized case report form for each visit.

### Study Procedures

**Fig 2:**
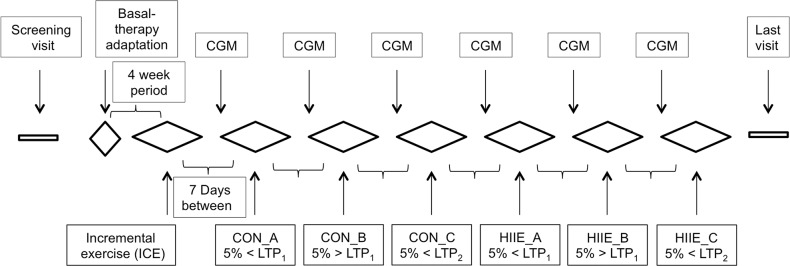
Timeline chart of the study procedure. CGM: Continuous glucose monitoring system, ICE: Incremental exercise, LTP_1_: Lactate turn point 1, LTP_2_: Lactate turn point 2, CON: Moderate continuous exercise, HIIE: short high-intensity interval exercise.

The study consisted of 24 visits to the exercise physiology laboratory and the outpatient clinic of the Division of Endocrinology and Metabolism (Fig 2). Visits 1–10 were used for the adaptation of ultra-long-acting insulin, visit 11 was used for the incremental exercise test (IET) in the exercise physiology laboratory, visits 12, 14, 16, 18, 20, 22, 24 were used to equip subjects with the continuous glucose monitoring (CGM) system, visits 13, 15, 17 were used for the moderate continuous exercise tests (CON) in the laboratory, visits 19, 21, 23 were used for the short aerobic interval exercise tests (HIIE) in the laboratory, and visit 24 was the final visit with the study physician. Participant recruitment was performed from February 20, 2014 to February 28, 2014, with the last follow up (last visit) examination being on October 01, 2014. Participants were recruited via telephone calls, contact information was provided from a ‘volunteers participant list’ with T1DM patients. 8 subjects were screened for elligibilty, one subject missed the inclusion criteria and one subject did not perform the HIIE at the highest exercise intensity, and therefore was excluded from all calculations for the highest exercise intensity.

### Standard insulin use before the start of the study

Before adaptation to the new ultra-long-acting insulin, two patients used insulin Detemir (Levemir/ Novo Nordisk, Denmark) and five patients used insulin Glargine (Lantus/ Sanofi-Aventis, France) as basal therapy. All subjects were switched to ultra-long-acting insulin Degludec (Tresiba/ Novo Nordisk, Denmark) with a run in period of 4 weeks with 68 ± 13% of their usual long-acting insulin dose used before the study. Short-acting insulin therapy was not adapted for everyday conditions. Four participants used short-acting insulin Aspart (Novo Rapid, Novo Nordisk, Denmark) and three participants used short-acting insulin Lispro (Humalog/ Lilly, USA).

### Adaptation to ultra-long-acting insulin

Novel ultra-long-acting basal insulin Degludec (Tresiba/ Novo Nordisk, Denmark) with a once-daily dosage and a flat insulin profile with a low intra-individual variability [28] was applied. It is referred to be associated with the same risk of exercise-induced hypoglycemia as insulin Glargine (Lantus/ Sanofi-Aventis, France) during low-intensity exercise [29].

The usual daily basal insulin dose was determined over the last four weeks prior to the start of this trial. Once per day in the evening, insulin Degludec was subcutaneously injected by the subject at home, with a starting dosage of 70% of total daily basal insulin dose. A run in period of four weeks assured an optimal setting for the basal insulin dose.

### Carbohydrate intake and short-acting insulin reductions

Each participant obtained a standardized meal before IET, HIIE, and CON. It was calculated from the amount of consumed carbohydrates at breakfast and then averaged over the last four weeks prior to the start of the study. Standardized fluid clinical nutrition consisted of 39% carbohydrates, 36% proteins and 25% fat (Fortimel Extra, Nutricia GmbH, Germany). Carbohydrate supplements and short-acting insulin injection were administered four hours before all exercise tests to avoid short-acting insulin impact during exercise as much as possible. Immediately after exercise, subjects injected the same short-acting insulin dose and ingested the same standardized carbohydrate amount as before testing. They were requested to continue usual injection rates according to the calculated carbohydrate factor (CarbF = (5.7 x weight (kg))/total daily dose) [30].

Short-acting insulin dose was reduced by 40% of the regular dose for the IET. For HIIE and CON, short-acting insulin dose reduction was depended on P_mean_ and set at 25% for A, 50% for B, and 75% for C, respectively. Consequently, short-acting insulin dose was 3 ± 1 U before IET, 4 ± 1 U before HIIE and CON for exercise intensity A, 2 ± 1 U for B, and 1 ± 0.5 U for C. The insulin administration was combined with an average carbohydrate ingestion of 59 ± 8 g.

### Exercise test protocols

#### Incremental exercise test (IET)

All participants performed IET until exhaustion [24]. At the beginning of the test, subjects had to sit quietly on the cycle ergometer for 3 min (0 W) before they started the warm-up period of 3 min with cycling at a workload of 40 W. Then, the workload was increased by 20 W every minute until volitional exhaustion. Finally, 3 min active recovery at 40 W followed by 3 min passive recovery (0 W) were conducted on the cycle ergometer. LTP_1_, LTP_2_, and P_max_ were determined in order to prescribe the exercise intensities for HIIE and CON individually.

#### Continuous exercise (CON)

Three CON tests were separated by one week. CON was performed for a total duration of 30 min at three different target workloads (= P_mean_) which represented intensities of common daily activities and were suggested to yield different responses [14]: 5% of P_max_ from IET below P_LTP1_ (A), 5% of P_max_ from IET above P_LTP1_ (B), and 5% of P_max_ from IET below P_LTP2_ (C). These target workloads represent intensities such as common low-intensity physical activity and occupational work for several hours (A), moderate steady state walking or low-intensity running or cycling (B), and strenuous exercise near the maximal lactate steady state (LaSS_max_) for a limited duration (C). Each test started with a 3 min resting period (sitting quietly on the cycle ergometer (0 W), followed by a 3 min warm-up at 40 W and a stepwise intensity increase by 20 W/min (to control for day-to-day variations in exercise response) until the final target workload was reached. This target workload was maintained for 30 min. Active and passive recovery periods of 3 min each were the same as in IET.

#### Aerobic high-intensity interval exercise (HIIE)

Three HIIE tests were separated by one week. Mean intensities (A, B, C) and total duration (30 min) were the same as for CON. The initial resting period as well as the following warm-up phase were equal to CON. Then, the specific short interval protocol started. For all mean intensities, P_peak_ was set at P_max_ from IET, and a short peak workload duration (t_peak_) of 20 s was applied. For A, recovery duration (t_rec_) was 120 s (work to rest ratio was 1:6); for B, t_rec_ was 60 s (work to rest ratio was 1:3); and for C, t_rec_ was 20 s (work to rest ratio was 1:1). The load for active recovery for A, B, and C were calculated according to the formula: P_mean_ = (P_peak_ * t_peak_ + P_rec_ * t_rec_) / (t_peak_ + t_rec_) [14] in order to guarantee the same mean loads for HIIE and CON and therefore comparable conditions between both exercise modes. Each HIIE protocol started with the recovery period and ended with a high-intensity peak workload phase. Finally, 3 min active recovery at 40 W and 3 min passive recovery (0 W) were conducted as in IET and CON.

### Measurements

Participants were equipped with a CGM system (Guardian, REAL-Time System, Medtronic, Minnesota, USA) and became familiar with its use. The sensor was inserted subcutaneously 24 h before testing to control for pre- and post-exercise glycemic response for a period of 48 h.

In all tests, capillary blood samples were taken from the ear lobe at rest, every 5 min during each specific exercise protocol, as well as at the end of the active and passive recovery periods, to determine lactate and BG concentrations by means of a fully enzymatic-amperometric method (Biosen S-line, EKF Diagnostics, Germany). Both LTP_1_ and the LTP_2_ were determined from the IET by means of a computer-based linear regression break point analysis, as shown earlier [24]. LTP_1_ was defined as the first increase in blood lactate concentration above baseline, and LTP_2_ was defined as the second abrupt increase of blood lactate between LTP_1_ and P_max_. Pulmonary gas exchange variables were collected continuously during all tests by breath-by-breath measurement and averaged over 5 s (ZAN 600, ZAN, Germany). Heart rate was measured continuously via chest belt telemetry during all tests and also averaged over 5 s (PE 4000, Polar Electro, Finland). A 12-lead ECG and blood pressure measurements (every 2 min) were obtained in all tests for cardiac monitoring. Adrenaline, noradrenaline, dopamine, cortisol, glucagon, and insulin-like growth factor-1 (IGF-1) were determined from venous blood samples obtained from a cubital vein immediately before exercise and after 15 and 30 min during HIIE and CON for A, B, and C. Adrenaline, noradrenaline and dopamine were quantified by RIA (DRG Diagnostics, USA), glucagon by RIA (ICN, USA) and cortisol and IGF-1 by CLIA (CENTAUR, Siemens Healthcare Diagnostics, USA).

Additionally, in order to prospectively determine a critical time limit to reach 50% of baseline glucose in HIIE vs. CON, the BG decrease was extrapolated in both exercise modes.

### Statistical analysis

A repeated-measures ANOVA design (number of repetitions = 4) was calculated a priori with a medium effect size of 0.5 and an alpha-error of 0.05 based on pilot data. Correlations between repetitions were assumed to equal 0.5. With a sample size of 6 patients, the achieved power (beta-1) is greater than 0.95 and is therefore appropriate for a high risk study. In case of a dropout we decided to conduct our study with 7 subjects. All data were normally distributed (Shapiro-Wilk normality test). Descriptive statistics including mean and standard deviation (SD) was performed for participants’ anthropometric data, performance characteristics and diabetes-specific data. Relationships between variables were performed by a Pearson`s correlation coefficient analysis. For analysis of BG decrease and hormone response from baseline to the end of exercise and CGM post-exercise glucose levels over 24 h, an analysis of variance (ANOVA) for repeated measures with paired t-test was used. For the comparison of HIIE and CON for BG decrease, CGM values, carbohydrate utilization, and the response of hormones, blood lactate, RER, and heart rate, a paired t-test was applied. All statistics were performed with a standard software package Prism Software version 4.0 (GraphPad, USA).

## Results

### Exercise performance data

The incremental exercise test revealed the participants’ VO_2max_ at 3.89 ± 0.74 l.min^-1^. P_LTP1_ was found at 82.1 ± 20.2 W, P_LTP2_ at 192.1 ± 33.6 W, and P_max_ at 284.3 ± 43.1 W.

### Blood lactate (La)

**Fig 3:**
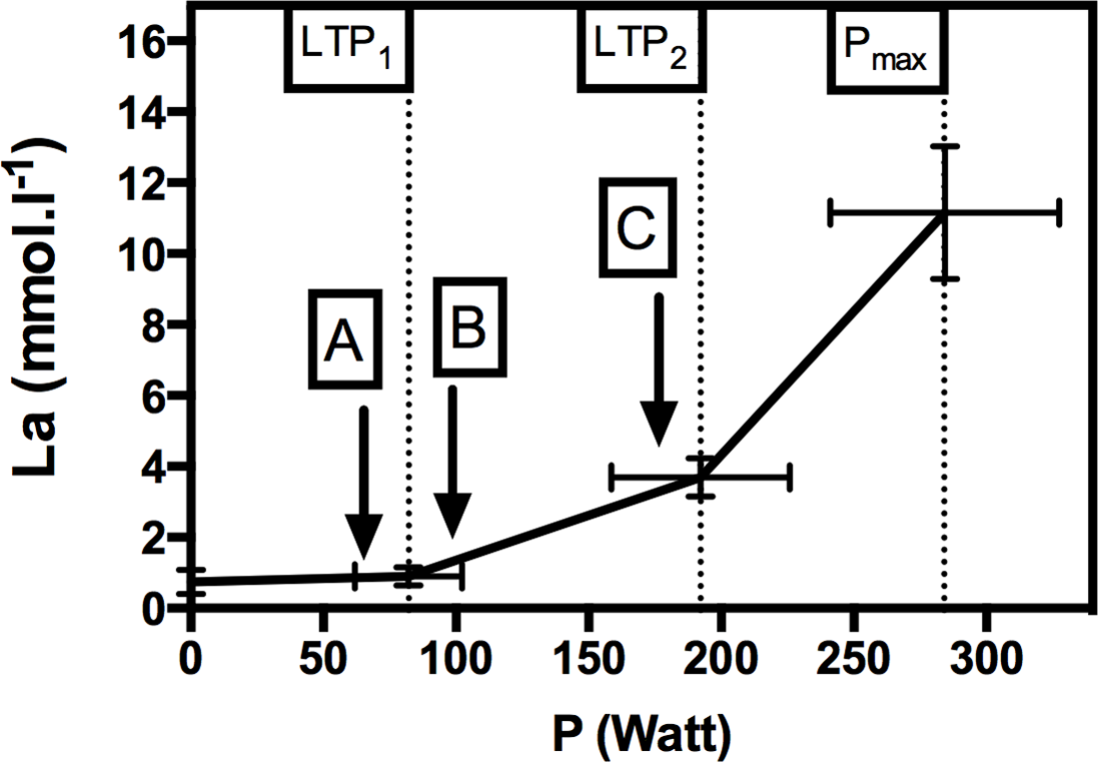
Blood lactate (La) performance curve during the incremental exercise test. A, B, and C represent the target mean loads for both continuous exercise and high-intensity interval exercise. LTP_1_: first lactate turn point; LTP_2_: second lactate turn point; P_max_: maximal power output. Values are given as mean and SD.

As shown in Fig 3, the blood lactate response during incremental exercise clearly showed the three phases of metabolism with two corresponding turn points (LTP_1_, LTP_2_). No significant increase was found from resting levels (0.75 ± 0.35 mmol.l^-1^) to LTP_1_ (0.90 ± 0.26 mmol.l^-1^) (p = 0.381). However, a significant increase was found from LTP_1_ up to LTP_2_ (3.69 ± 0.54 mmol.l^-1^) (p < 0.001) and from LTP_2_ up to P_max_ (11.16 ± 1.88 mmol.l^-1^) (p < 0.001). LTP_1_ and LTP_2_ were not significantly different from gas exchange thresholds (data not shown).

**Fig 4:**
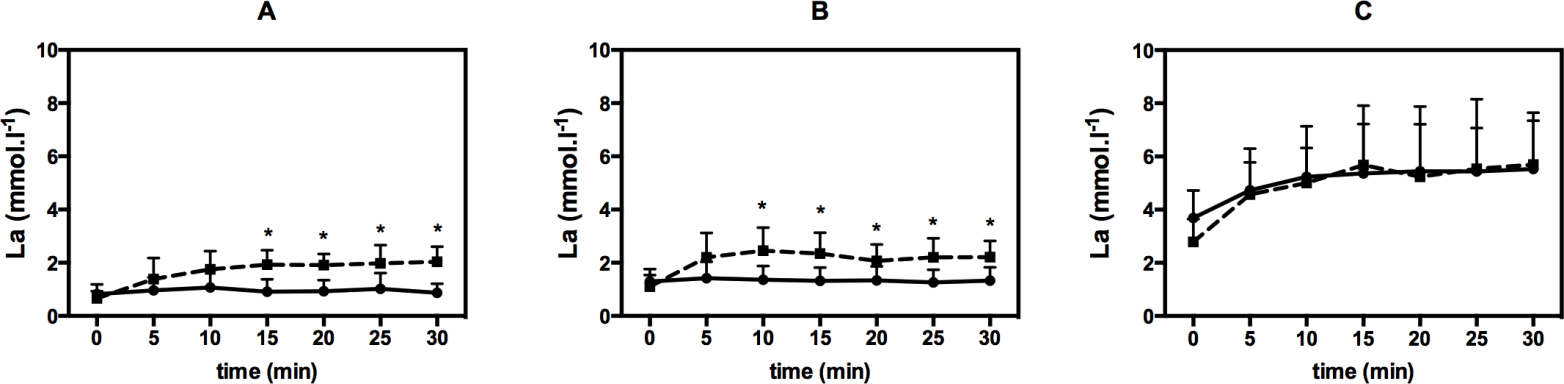
Comparison of blood lactate response (La) during high-intensity interval exercise (dotted line) vs. continuous exercise (full line) for mean exercise intensities A, B, and C. Values are given as mean and SD. “*” represents significance.

Also during CON, the metabolic response was shown to be related to LTP_1_ and LTP_2_ and gave a lactate steady state (LaSS) slightly below (A) and above resting values (B) and a La response near the maximal LaSS (C) with significantly elevated values compared to A and B (p < 0.001). As expected, a LaSS at all mean intensities was found in short HIIE as well (Fig 4).

In comparison of both exercise regimes, mean La values were significantly higher in HIIE vs. CON for A (1.67 ± 0.50 vs. 0.96 ± 0.41 mmol.l^-1^, p = 0.006) and for B (2.08 ± 0.64 vs. 1.33 ± 0.48 mmol.l^-1^, p = 0.004). No significant difference for La was found for C (4.93 ± 1.80 vs. 4.75 ± 1.53 mmol.l^-1^) (p = 0.418) (Fig 4).

### Blood glucose (BG) decrease

During and after all test sessions, no hypoglycemic event (defined as a BG concentration lower than 3.3 mmol.l^-1^) occurred. All participants maintained a BG steady state during IET after a 40% short-acting insulin reduction and with no significant difference between the start (10.58 ± 3.42 mmol.l^-1^) and the end (10.24 ± 3.48 mmol.l^-1^) of exercise (p > 0.857).

Starting BG for HIIE was 11.06 ± 1.31 mmol.l^-1^ (A), 12.31 ± 2.36 mmol.l^-1^ (B), and 12.24 ± 2.88 mmol.l^-1^ (C). Starting BG for CON was found at 10.42 ± 2.01 mmol.l^-1^ (A), 12.78 ± 3.76 mmol.l^-1^ (B), and 12.84 ± 3.21 mmol.l^-1^ (C).

**Fig 5:**
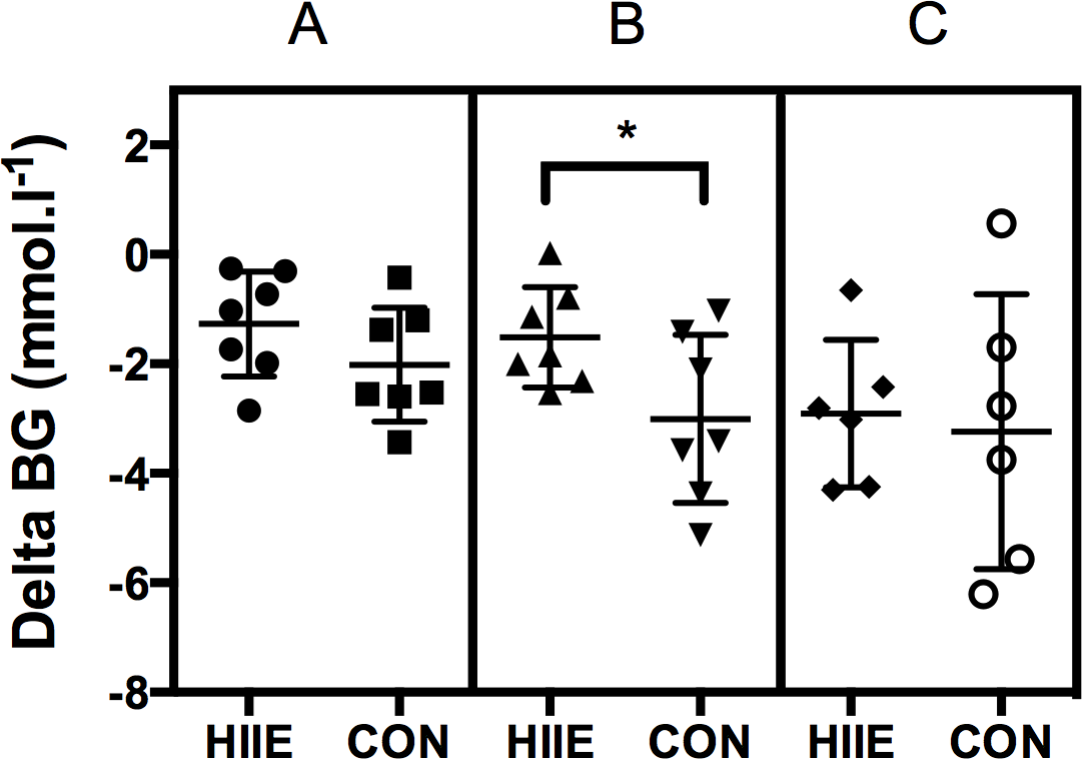
Comparison of delta blood glucose (BG) during high-intensity interval exercise vs. continuous exercise for mean exercise intensities A, B, and C. Values are given as mean and SD. “*” represents significance.

**Fig 6:**
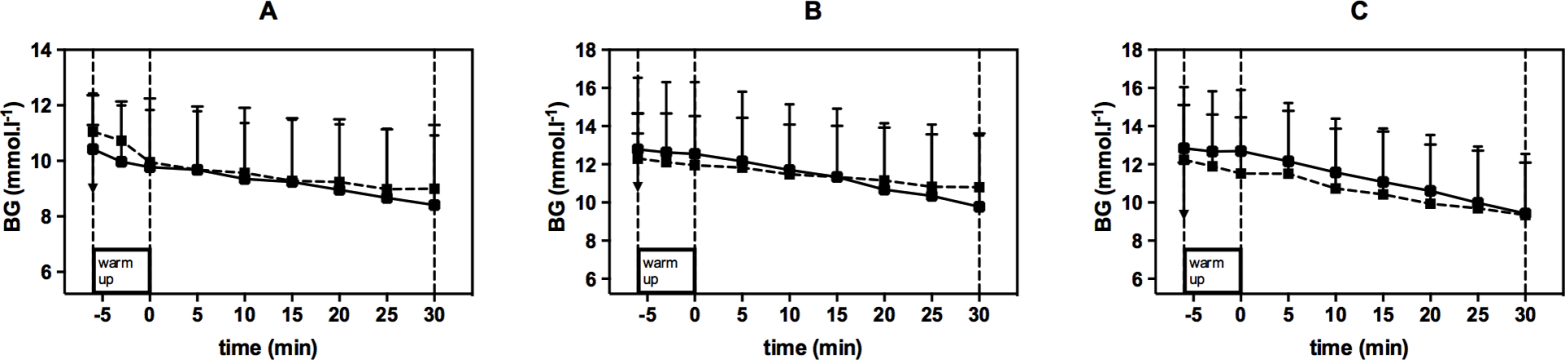
Comparison of blood glucose (BG) decrease during high-intensity interval exercise (dotted line) vs. continuous exercise (full line) for exercise intensities A, B, and C. Values are given as mean and SD.

BG decreased linearly, but not significantly (p = 0.132), from the start to the end of exercise. In comparison of both exercise modes, BG decrease was significantly smaller during HIIE vs. CON for B (1.51 ± 0.92 vs. 3.00 ± 1.54 mmol.l^-1^, p = 0.024) and smaller by trend for A (1.27 ± 0.96 vs. 2.01 ± 1.04 mmol.l^-1^, p = 0.244) and C (2.91 ± 1.35 vs. 3.42 ± 2.34 mmol.l^-1^, p = 0.573) (Figs 5 and 6). The BG decrease, expressed as percentage from starting BG, was also significantly lower at B (p = 0.011) and lower by trend at A (p = 0.235) and C (p = 0.845) during the intervals compared to continuous exercise. In addition, we found that BG decrease significantly correlated with the mean exercise intensity (p = 0.008).

Decreases of BG levels were slightly, but not significantly correlated with carbohydrate utilization (p = 0.152), except for C in HIIE (p = 0.015) which was calculated indirectly from respiratory data [31]. In HIIE, carbohydrate utilization was found to be 1.26 ± 0.27 g.min^-1^ (A), 1.59 ± 0.38 g.min^-1^ (B), and 3.20 ± 0.53 g.min^-1^ (C). In CON, carbohydrate utilization was shown to be 1.08 ± 0.39 g.min^-1^ (A), 1.28 ± 0.42 g.min^-1^ (B), and 3.07 ± 0.44 g.min^-1^ (C).

### Late glycemic onset (CGM data)

In both short HIIE and CON, no hypoglcemia occurred for 24 h after exercise. Mean glucose levels during 24 h post-exercise were found to be 9.94 ± 2.00 mmol.l^-1^ (A), 8.77 ± 2.78 mmol.l^-1^ (B), and 8.55 ± 1.94 mmol.l^-1^ (C) in HIIE. Mean values for CON were 8.21 ± 1.39 mmol.l^-1^ (A), 9.82 ± 1.67 mmol.l^-1^ (B), and 9.38 ± 3.27 mmol.l^-1^ (C). No significant differences were found in mean values between HIIE and CON (p = 0.833).

### Hormones

**Fig 7:**
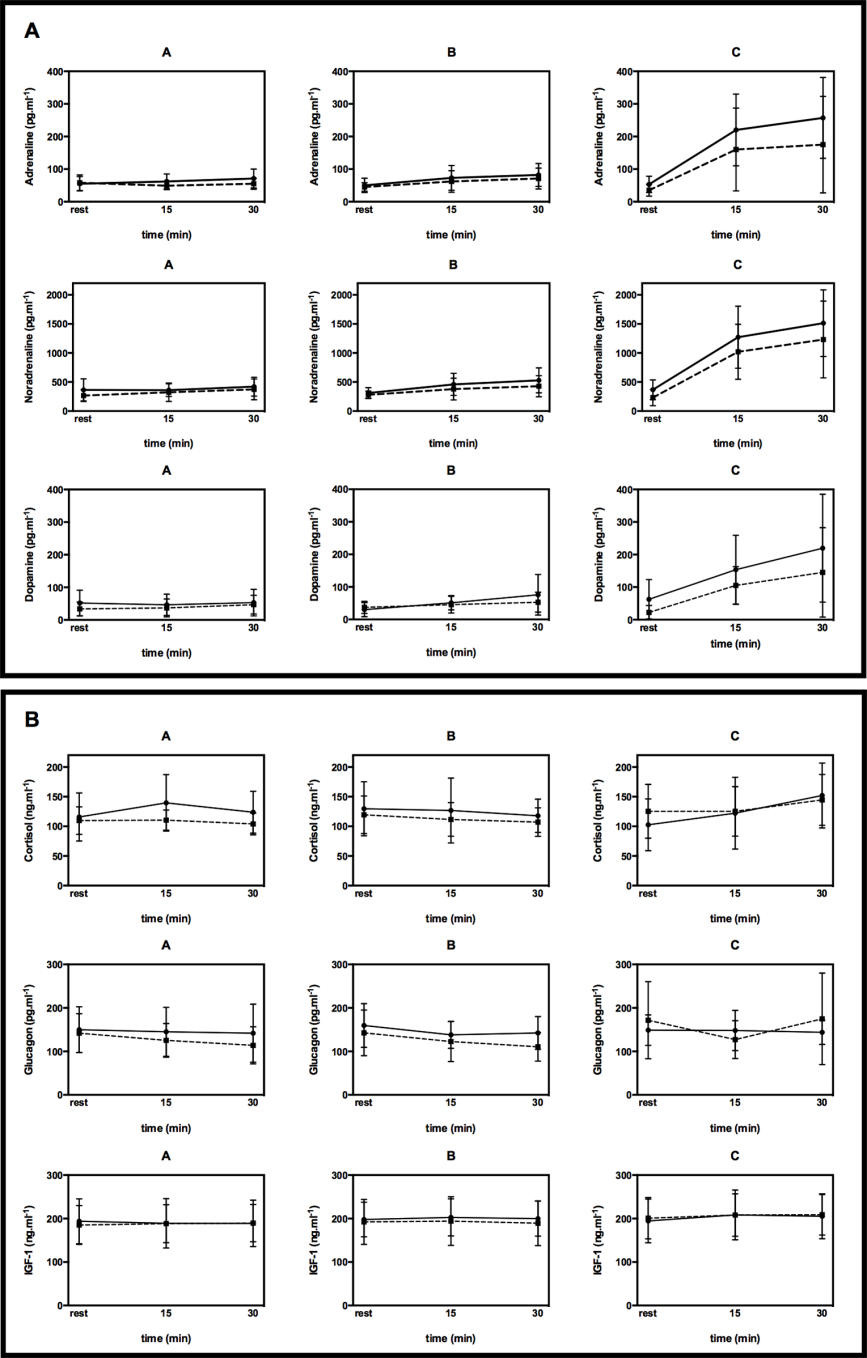
Comparison of hormone response during high-intensity interval exercise (dotted line) vs. continuous exercise (full line) for mean exercise intensities A, B, and C. (A) Catecholamine response. Values are given as mean and SD. (B) Glucagon, cortisol and IGF-1. IGF-1: insulin-like growth factor-1. Values are given as mean and SD.

Dependent on the mean exercise intensity, baseline values of adrenaline (p = 0.001), noradrenaline (p < 0.001), and dopamine (p = 0.003) significantly increased up to the end of exercise in both HIIE and CON. Slight, but not significant, increases were found for cortisol (p = 0.082) and IGF-1 (p = 0.084) over time in HIIE and CON, but not for glucagon (p = 0.153) (Fig 7).

Interestingly, no significant difference in the mean values of adrenaline (p = 0.241), noradrenaline (p = 0.153), dopamine (p = 0.552), cortisol (p = 0.398), IGF-1 (p = 0.511) and glucagon (p = 0.447) was found between the exercise modes for A, B, and C, even despite higher peak workloads in HIIE (Fig 7).

### Respiratory exchange ratio (RER)

**Fig 8:**
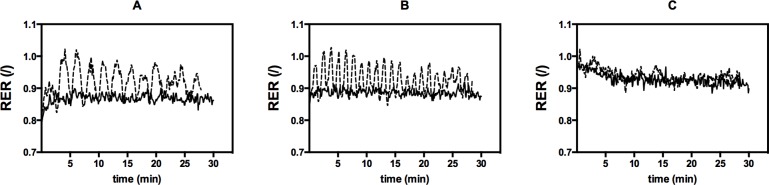
Comparison of the respiratory exchange ratio (RER) response during high-intensity interval exercise (dotted line) vs. continuous exercise (full line) for mean exercise intensities A, B, and C. Values are given as mean.

RER values were significantly related to mean exercise intensity (p = 0.009) as expected (Fig 8). Displaying similar results as mean La, mean RER values were also significantly higher during HIIE vs. CON for A (0.92 ± 0.03 vs. 0.87 ± 0.03, p = 0.003) and B (0.93 ± 0.03 vs. 0.89 ± 0.03, p = 0.0034), but no significant difference was found for C (0.94 ± 0.03 vs. 0.93 ± 0.03, p = 0.091). However, peak RER values during the high-intensity work bouts were higher for A and B than for C (Fig 8).

### Heart rate (HR)

**Fig 9:**
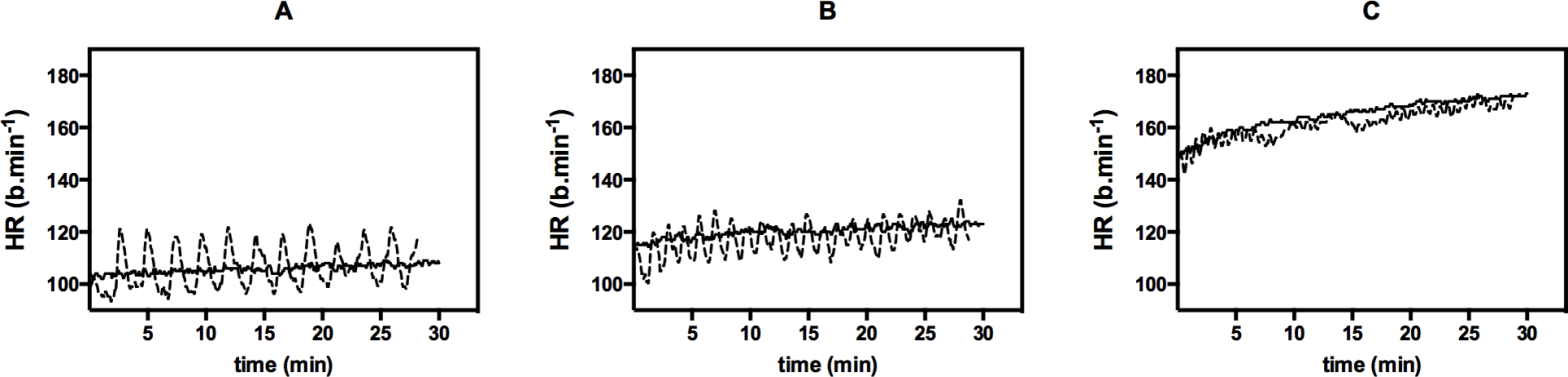
Comparison of heart rate (HR) response during high-intensity interval exercise (dotted line) vs. continuous exercise (full line) for mean exercise intensities A, B, and C. Values are given as mean.

Mean heart rate expectedly increased depending on exercise intensity (p < 0.001) in both HIIE and CON for all mean loads (Fig 9). No significant difference for mean HR was found between exercise modes at A, B, and C (HIIE vs. CON: 106.4 ± 11.1 vs. 105.8 ± 7.0 b.min^-1^, p = 0.845 (A), 117.5 ± 9.3 vs. 120.2 ± 7.6 b.min^-1^, p = 0.324 (B), 161.8 ± 8.8 vs. 164.7 ± 9.6 b.min^-1^, p = 0.186 (C)).

## Discussion

Our methodological approach for exercise prescription was completely novel for patients with T1DM. First, short HIIE and CON were matched for the same mean load (P_mean_) and duration, which is supported by standard physiological measurements such as blood lactate, heart rate, and gas exchange data. Therefore, both exercise modes were comparable.

Second, the intensities for HIIE (peak workload, recovery workload, and mean load) and CON (target workload = mean load) were set with respect to objective and individual submaximal (LTP_1_, LTP_2_) and maximal markers (P_max_) from the IET [24], in order to prescribe the exercise intensity as individually and accurately as possible. This exercise prescription model is metabolically justifiable and prevents unpredictable and heterogeneous responses across subjects [13]. This could be clearly shown for patients with T1DM in this study supporting our methodological approach.

In addition, new ultra-long-acting insulin Degludec (Tresiba/ Novo Nordisk, Denmark) was used, and short-acting insulin was reduced depending on mean exercise intensity.

### Risk of hypoglycemia, BG decrease during exercise, and late onset (interstitial) glucose

The present study revealed that for all three mean intensities, neither CON nor short HIIE induced hypoglycemic events during and over a 24 h period after exercise in type 1 diabetic subjects. This is in line with results from Rabasa-Lhoret et al. [4] who found a considerably reduced risk of hypoglycemia during and after CON after the same exercise intensity-dependent short-acting insulin reduction as in our study.

In order to estimate the risk of hypoglycemia during exercise durations longer than 30 min, we calculated the time range for BG decrease from starting level to 50% of the starting level by linear extrapolation. This time range was mean intensity-dependent (significantly longer for A and B vs. C, p < 0.001) and significantly longer in HIIE vs. CON (p = 0.016): 142.0 vs. 95.0 min (A), 140.0 vs. 66.0 min (B), and 64.5 vs. 57.0 min (C). Therefore, it is suggested that aerobic short HIIE can be sustained for a longer time without risk of hypoglycemia than CON. However, extrapolated data have to be interpreted with care as starting glucose levels influence this time range, and the application of a linear regression has some limits.

The BG decrease during exercise in our study was significantly lower for B and lower by trend for A and C during HIIE vs. CON, supporting the results of other studies [17, 19, 20, 28]. Given that meals, basal insulin, and short-acting insulin reduction were standardized and that the hormone responses were similar in both exercise modes, the question arises, why BG decrease was smaller in short HIIE than in CON. Guelfi et al. [32] could clearly show that during exercise, the decline in glycemia was smaller in very short HIIE (t_peak_ = 4 s) when compared to CON. This was due to a greater increment in endogenous glucose production, despite an earlier increase in glucose utilization which was attenuated at the end of HIIE. In line with that, Purdon et al. [33] and Sigal et al. [34] pointed out that the increase in endogenous glucose production was disproportionately greater than the increase in glucose utilization during exercise. Guelfi et al. [32] argued that during HIIE, the greater rise in glucose production was mainly due to an elevated distribution of noradrenaline. The earlier increase in glucose utilization during HIIE was related to the higher peak workload and primarily provided by muscle glycogen breakdown. However, the authors used a test design with a higher total work in HIIE compared to CON. This difference in P_mean_ may explain the higher noradrenaline distribution during HIIE, which was not found in our study.

In addition, we suggest that during the high-intensity peak workload phases during HIIE, with a high glucose turnover rate, glucose for energy supply is provided from intracellular glycogen stores rather than from blood glucose. Furthermore, during recovery phases of HIIE, intracellular La produced during the peak workload phases may be oxidized rather than blood glucose (glucose sparing effect) [35]. This suggestion is supported by the fact that high peak RER values were found during HIIE for A and B whereas blood La values remained low. An inhibition of glycolysis during HIIE is due to lowered pH values and/or an accumulation of citrate in the cytoplasm during recovery; however, this seems implausible since we see the higher carbohydrate consumption in HIIE for A, B, and C.

In contrast to the aforementioned studies, Iscoe and Riddell [16] did not find a difference in the decrease of plasma glucose and whole blood glucose during HIIE vs. CON in T1DM patients.

The interstitial (late onset) glucose levels measured for 24 h post-exercise were not significantly different in short HIIE vs. CON after all tests in our study. The risk of post-exercise hypoglycemia was, therefore, similar in both exercise modes (neither HIIE nor CON led to hypoglycemic events). Our findings are contrary to the results of Iscoe and Riddell [16] who found higher nocturnal glucose values in the HIIE group vs. the CON group. Guelfi et al. [32] stated that during HIIE, both endogenous glucose production and glucose utilization declined earlier after exercise and then stayed slightly higher for two hours compared to CON.

The question arises, if post-exercise glucose utilization is provided rather by intracellular glycogen stores of the working muscle or by blood glucose. Furthermore, it is not clear to what extent glucose, synthesized by lactate (produced in the working skeletal muscle) via gluconeogenesis, may contribute to reducing the risk of hypoglycemia for 24 h after exercise.

### Hormones

No significant difference in the acute hormone response was found between aerobic short HIIE and moderate CON for any mean load, despite significantly higher peak workloads in HIIE. In both short HIIE and CON, hormonally and metabolically balanced conditions were yielded. Our results are in line with Iscoe and Riddell [16] who also found acute hormone responses that were not significantly different between short HIIE and CON when matched for P_mean_ and duration.

### Metabolic and cardiorespiratory variables

For A and B, mean values for La were significantly higher in HIIE vs. CON, but quite low in both exercise modes. For C, La response was not significantly different between HIIE and CON reaching values up to 5 mmol.l^-1^, but metabolic steady state conditions could be maintained in both exercise regimes. However, the lactate response to HIIE is strongly related to the duration of the peak workload phases (t_peak_) [14, 36]. Therefore, it has to be pointed out that the results achieved from HIIE in this study are only valid for interval exercise with a short t_peak_ of 20 s. Campbell et al. [20] and Iscoe and Riddell [16] also revealed that the lactate response was higher in HIIE than in CON applying short intervals.

The mean RER response was similar to the response for La. However, the high peak RER values found for A and B were not reflected in the low blood lactate levels. It was clearly shown that for the high mean intensity (C), mean RER was not significantly different between the 20 s HIIE and CON with values far below 1.

The mean heart rate was positively correlated to the mean exercise intensity. Between HIIE and CON, mean HR was not significantly different as expected. HR peaks at A and B were higher in HIIE compared to CON because of fluctuations of the HR during the intervals, but still remained on a low level (< 130 b.min^-1^). The HR fluctuation became smaller with increased P_mean_ due to the shorter recovery phases. This supports the findings of Iscoe and Riddell [16].

A limitation of this study was that the performance of HIIE and CON were not randomized for safety reasons (starting with low mean workload and increasing mean load from test to test). However, this fact is not suggested influencing the results since subjects were trained. Therefore, training effects were unlikely. Another limitation was the low number of subjects for intention to treat reasons. One subject atypically showed no BG decrease during exercise. However, since this abnormal BG response was shown in each test, the subject was included in all calculations. Furthermore, measuring interstitial glucose by CGM is not as accurate as by means of venous blood samples.

In summary, it can be pointed out that both CON and short HIIE performed at different mean loads (ranging from intensities below P_LTP1_ up to slightly below P_LTP2_) are safe for T1DM patients if the pre- and post-exercise short-acting insulin dose is reduced depending on the mean exercise intensity and pre-exercise BG is high enough.

Further mechanistic models and sophisticated investigations such as tracer studies are required to understand the T1DM-specific acute physiological responses and cellular processes induced by high-intensity intermittent exercise. In addition, longitudinal studies involving patients with T1DM are of high relevance to investigate the middle- and long-term effects of different exercise training modes. Since the cardiac stress at the high mean intensity C was not elevated during the 20 s interval exercise compared to CON, and the HR peaks during HIIE at A and B remained on a low level, short HIIE is suggested to be safely applied also in the elderly, less trained subjects, and other groups of patients. The 4 x 4 min HIT [9,10] mode, which has been often applied in individuals with chronic diseases, is also required to be investigated in T1DM patients with respect to the acute physiological and hormonal responses and training adaptations which has not been done yet.

## Conclusions

The positive effects of physical activity and exercise training in patients with T1DM are evident. However, to balance long- and short-acting insulin administration, carbohydrate intake, as well as exercise intensity and duration is a crucial issue for type 1 diabetic subjects in order to avoid exercise-induced hypoglycemia. We could clearly show that this balance can be accomplished and that both aerobic short HIIE and CON matched for mean load and duration were safe. Importantly, an exercise intensity prescription that considers the individual first and second lactate turn point and the maximal power output was applied, and ultra-long-acting insulin Degludec (Tresiba/ Novo Nordisk, Denmark) was used. This study revealed that despite markedly higher peak workloads, aerobic HIIE with a t_peak_ of 20 s did neither yield higher risks of hypoglycemia during and after exercise nor higher acute responses for hormones compared to CON at different mean intensity domains. Short HIIE even led to a significantly (B) or by trend (A, C) smaller blood glucose decrease than CON. An extrapolation of BG decrease from baseline levels to 50% baseline BG clearly showed the slower decrease of blood glucose (smaller decrease per time) during HIIE. In addition, the study clearly showed that the BG decrease and therefore the risk of hypoglycemia during and for 24 hours after exercise is dependent on the mean exercise intensity and duration, whether this is achieved via HIIE or CON.

Based on our results, HIIE as well as CON can be recommended for patients with T1DM. Short-acting insulin is suggested to be reduced depending on mean exercise intensity in both exercise modes.

The revealed data allow the calculation of required BG baseline concentrations in T1DM patients for different mean exercise intensities, durations, and methods to avoid hypoglycemia and to minimize additional carbohydrate ingestion during exercise.

## Supporting Information

S1 Protocol Trial Protocol. (PDF)

S1 TREND Checklist TREND Checklist. (PDF)

## References

[1] Satoru K, Shiro T, Yoriko H, Kazuya F, Chika H, Hitoshi S, et al Association between physical activity and risk of all-cause mortality and cardiovascular disease in patients with diabetes: a meta-analysis. Diabetes Care. 2013; 36: 471–479. 10.2337/dc12-0783 23349151 PMC3554302

[2] Harding JL, Shaw JE, Peeters A, Cartensen B, Magliano DJ. Cancer risk among people with type 1 and type 2 diabtes: disentangling true associations, detection bias, and reverse causation. Diabetes Care. 2015; 38: 264–270. 10.2337/dc14-1996 25488912

[3] Chimen M, Kennedy A, Nirantharakumar K, Pang TT, Andrews R, Narendran P. What are the health benefits of physical activity in type 1 diabetes mellitus? A literature review. Diabetologia. 2012; 55: 542–551. 10.1007/s00125-011-2403-2 22189486

[4] Rabasa-Lhoret R, Bourque J, Ducros F, Chiasson J. Guidelines for premeal insulin dose reduction for postprandial exercise of different intensities and durations in type 1 diabetic subjects treated intensively with a basal-bolus insulin regimen (Ultralente-Lispro). Diabetes Care. 2001; 24: 625–630. 11315820 10.2337/diacare.24.4.625

[5] Billat LV. Interval training for performance: a scientific and empirical practice. Special recommendations for middle- and long-distance running. Part I: aerobic interval training. Sports Med. 2001; 31: 13–31. 11219499 10.2165/00007256-200131010-00002

[6] Helgerud J, Hoydal K, Wang E, Karlsen T, Berg P, Bjerkaas M, et al Aerobic high-intensity intervals improve VO2max more than moderate training. Med Sci Sports and Exerc. 2007; 39: 665–671. 17414804 10.1249/mss.0b013e3180304570

[7] Daussin FN, Zoll J, Dufour SP, Ponsot E, Lonsdorfer-Wolf E, Doutreleau S, et al Effect of interval versus continuous training on cardiorespiratory and mitochondrial functions: relationship to aerobic performance improvements in sedentary subjects. Am J Physiol Regul Integr Comp Physiol. 2008; 295: 264–272. 10.1152/ajpregu.00875.2007 18417645

[8] Meyer K, Samek L, Schwaibold M, Westbrook S, Hajric R, Beneke R, et al Interval training in patients with severe chronic heart failure–analysis and recommendation for exercise procedures. Med Sci Sports and Exerc. 1997; 29: 306–312. 9139168 10.1097/00005768-199703000-00004

[9] Wisloff U, Stoylen A, Loennechen JP, Bruvold M, Rognmo O, Haram PM, et al Superior cardiovascular effect of aerobic interval training versus moderate continuous training in heart failure patients: A randomized study. Circulation. 2007; 115: 3086–3094. 17548726 10.1161/CIRCULATIONAHA.106.675041

[10] Tjonna AE, Lee SJ, Rognmo O, Stolen TO, Bye A, Haram PM, et al Aerobic interval training versus continuous moderate exercise as a treatment for the metabolic syndrome: A pilot study. Circulation. 2008; 118: 346–354. 10.1161/CIRCULATIONAHA.108.772822 18606913 PMC2777731

[11] Smart NA, Dieberg G, Giallauria F. Intermittent versus continuous exercise training in chronic heart failure: A metaanalysis. Int J Cardiol. 2013;166: 352–358. 10.1016/j.ijcard.2011.10.075 22100179

[12] Iellamo F, Manzi V, Caminiti G, Vitale C, Castagna C, Massaro M, et al Matched dose interval and continuous exercise training induce similar cardiorespiratory and metabolic adaptations in patients with heart failure. Int J Cardiol. 2013; 167: 2561–2565. 10.1016/j.ijcard.2012.06.057 22769574

[13] Tschakert G, Kroepfl J, Mueller A, Moser O, Groeschl W, Hofmann P. How to regulate the acute physiological response to “aerobic" high-intensity interval exercise. J Sports Sci Med. 2015; 14: 29–36. 25729286 PMC4306779

[14] Tschakert G, Hofmann P. High-intensity intermittent exercise: methodological and physiological aspects. Int J Sports Physiol Perform. 2013; 8: 600–610. 23799827 10.1123/ijspp.8.6.600

[15] Keteyian SJ. Swing and a miss or inside the park home run: Which fate awaits high intensity exercise training? Circulation. 2012; 126, 1431–1433. 10.1161/CIRCULATIONAHA.112.129171 22879368

[16] Iscoe KE, Riddell MC. Continuous moderate-intensity exercise with or without intermittent high-intensity work: effects on acute and late glycaemia in athletes with type 1 diabetes mellitus. Diabetic Med. 2011; 28: 824–832. 10.1111/j.1464-5491.2011.03274.x 21388440

[17] Bussau VA, Ferreira LD, Jones TW, Fournier PA. The 10-s maximal sprint: a novel approach to counter an exercise-mediated fall in glycemia in individuals with type 1 diabetes. Diabetes Care. 2006; 3: 601–606. 10.2337/diacare.29.03.06.dc05-1764 16505513

[18] Guelfi KJ, Jones TW, Fournier PA. Intermittent high-intensity exercise does not increase the risk of early postexercise hypoglycemia in individuals with type 1 diabetes. Diabetes Care. 2005; 28: 416–418. 15677802 10.2337/diacare.28.2.416

[19] Maran A, Pavan P, Bonsembiante B, Brugin E, Ermolao A, Avogaro A, et al Continuous glucose monitoring reveals delayed nocturnal hypoglycemia after intermittent high-intensity exercise in nontrained patients with type 1 diabetes. Diabetes Technol Ther. 2010; 12: 763–768. 10.1089/dia.2010.0038 20807120

[20] Campbell MD, West DJ, Bain SC, Kingsley MIC, Foley P, Kilduff L, et al Simulated games activity vs continuous running exercise: a novel comparison of the glycemic and metabolic responses in T1DM patients. Scand J Med Sci Sports. 2014 3 4 10.1111/sms.12192 24593125

[21] Hofmann P, von Duvillard SP, Seibert FJ, Pokan R, Wonisch M, Lemura LM, et al %HR_max_ target heart rate is dependent on heart rate performance curve deflection. Med Sci Sports Exerc. 2001; 33: 1726–1731. 11581558 10.1097/00005768-200110000-00017

[22] Wonisch M, Hofmann P, Fruhwald FM, Kraxner W, Hödl R, Pokan R, et al Influence of beta-blocker use on percentage of target heart rate exercise prescription. Eur J Cardiovasc Prev Rehab. 2003; 10: 296–301. 10.1097/00149831-200308000-00013 14555886

[23] Scharhag-Rosenberger F, Meyer T, Gäßler N, Faude O, Kindermann W. Exercise at given percentages of VO_2max_: heterogeneous metabolic response between individuals. J Sci Med Sport. 2010; 13: 74–79. 10.1016/j.jsams.2008.12.626 19230766

[24] Hofmann P, Tschakert G. Special needs to prescribe exercise intensity for scientific studies. Cardiol Res Prac, 2010 11 9 Article ID 209302, 10 pages. 10.4061/2011/209302 PMC3010619 21197479

[25] Davis HA, Bassett J, Hughes P, Gass GC. Anaerobic threshold and lactate zurnpoint. Eur J Appl Physiol Occup Physiol. 1983; 50: 383–392. 6683162 10.1007/BF00423244

[26] Skinner JS, McLellan TH. The Transition from aerobic to anaerobic metabolism. Res Q Exerc Sport. 1980; 51: 234–248. 7394286 10.1080/02701367.1980.10609285

[27] Brooks GA. Cell-cell and intracellular lactate shuttles. J Physiol. 2009; 587: 5591–5600. 10.1113/jphysiol.2009.178350 19805739 PMC2805372

[28] Heise T, Hermanski L, Nosek L, Feldman A, Rasmussen S, Haahr H. Insulin degludec: Four times lower pharmacodynamic variability than insulin glargine under steady-state conditions in type 1 diabetes. Diabetes Obes Metab. 2012; 14: 859–864. 10.1111/j.1463-1326.2012.01627.x 22594461

[29] Heise T, Zijlstra E, Nosek L, Bracken R, Haahr HL, Roepstorff C, et al. Similar risk of exercise-related hypoglycaemia for insulin degludec compared to insluin glargine in patients with type 1 diabetes (poster no. PD-0791). World Diabetes Congress; 2–6 Dec 2013; Melbourne. 10.1111/dom.12588 PMC5063138 26450456

[30] Walsh J, Roberts R, Bailey T. Guidelines for optimal bolus calculator settings in adults. J Diabetes Sci Techno. 2011; 5: 129–135. 10.1177/193229681100500118 PMC3045234 21303635

[31] Jeukendrup AE, Wallsi GA. Measurement of substrate oxidation during exercise by means of gas exchange measruements. Int J Sports Med. 2005; 26: 28–33. 10.1055/s-2004-830512 15702454

[32] Guelfi KJ, Ratnam N, Smythe GA, Jones TW, Fournier PA. Effect of intermittent high-intensity compared with continuous moderate exercise on glucose production and utilization in individuals with type 1 diabetes. Am J Physiol Endocrinol Metab. 2007; 292: 865–870. 10.1152/ajpendo.00533.2006 17339500

[33] Purdon C, Brousson M, Nyveen SL, Miles PD, Halter JB, Vranic M, et al The roles of insulin and catecholamines in the glucoregulatory response during intense exercise and early recovery in insulindependent diabetic and control subjects. J Clin Endocrinol Metab. 1993; 76: 566–573. 8445012 10.1210/jcem.76.3.8445012

[34] Sigal RJ, Purdon C, Fisher SJ, Halter JB, Vranic M, Marliss EB. Hyperinsulinemia prevents prolonged hyperglycemia after intense exercise in insulin-dependent diabetic subjects. J Clin Endocrinol Metab. 1994; 79: 1049–1057. 7962273 10.1210/jcem.79.4.7962273

[35] Emhoff CA, Messonnier LA, Horning MA, Fattor JA, Carlson TJ, Brooks GA. Direct and indirect lactate oxidation in trained and untrained men. J Appl Physiol (1985). 2013; 115: 829–838. 23788576 10.1152/japplphysiol.00538.2013 PMC8846964

[36] Astrand I, Astrand PO, Christensen EH, Hedman R. Intermittent muscular work. Acta Physiol Scand. 1960; 48: 448–453. 13794890 10.1111/j.1748-1716.1960.tb01879.x

