# Supplementary material for: Effects of High-Intensity Interval Exercise versus Moderate Continuous Exercise on Glucose Homeostasis and Hormone Response in Patients with Type 1 Diabetes Mellitus Using Novel Ultra-Long-Acting Insulin
Source: PLoS One. 2015 Aug 28;10(8):e0136489. doi: 10.1371/journal.pone.0136489 (PMC4552855; doi:10.1371/journal.pone.0136489)
Supplement: S1 Protocol — (PDF) [file pone.0136489.s001.pdf]

Medizinische Universität Graz

EXERCISE AND BLOOD GLUCOSE LEVELS IN PATIENTS WITH TYPE I DIABETES – A PILOT STUDY

Blutzuckereinstellung bei Diabetes mellitus Typ I in Abhängigkeit von unterschiedlichen Belastungen – eine Pilotstudie

Principal Investigator: Prof. Dr. Thomas Pieber

Co-Principal Investigator: Ao. Univ. Prof. Mag. Dr. Peter Hofmann, FASCM

Version 2.0
09.12.2013

Table of contents

1. Introduction ... 3
1.1. Pathophysiology of type 1 diabetes mellitus ... 3
1.2. Physiology of type 1 diabetes mellitus in sports ... 3
1.3. Adaptation of carbohydrate intake and insulin injection before an exercise in type 1 diabetes mellitus ... 4
2. Pilot study ... 5
3. Study goal and hypothesis ... 6
4. Methods ... 7
4.1. General study design ... 7
4.2. Subjects recruitment ... 8
4.3. Screening visit ... 9
4.4. Adaptation of basal insulin degludec (Tresiba®, Novo Nordisk) ... 9
4.5. Calculation of carbohydrate factor and correction factor ... 9
4.6. Install of CGMS and edition carbohydrate supplement ... 10
4.7. Maximal incremental cycle ergometer exercise test ... 10
4.8. Constant load test ... 11
4.9. High intensity intermittent exercise tests ... 12
4.10. Measurements ... 14
4.10.1. Anthropometric data and TDD ... 14
4.10.2. Blood glucose, gas exchange, heart rate variables and lactate ... 14
4.10.3. Continuous glucose montoring system (CGMS) ... 14
4.10.4. Hormones ... 14
5. Statistics ... 16
6. Time table for subjects ... 17
7. Dissemination strategies and potential impact ... 18
8. Cooperation partners and resources ... 19
9. References ... 21

1. Introduction

1.1. Pathophysiology of type 1 diabetes mellitus

Type 1 diabetes mellitus (T1DM) is an metabolic disease with insulin deficiency and a dysfunctional release of counter regulatory hormone glucagon (Oyer 2013; Cryer 2012; Younk et al. 2011). In the pathogenesis of T1DM autoantibodies to insulin (IAA), glutamatdecarboxylase antibodies (GADA), islet antigen-2 (IA-2A) and zinc transporter 8 (ZnT8A) (Larsson et al. 2013; Han et al. 2013; Long et al. 2012) can be detected. In absence of endogen produced insulin the translocating glucose transporters (GLUT) cannot be activated (excepted insulin insensitive GLUT-2 in liver, β-cells of pancreas, kidneys and small intestine and GLUT-4 while muscle contraction) (Jensen & Richter 2012). The GLUT translocate to the cell surface and import glucose into the cell for metabolism or glycogen storage by glucose-6-phosphatase (G-6-P) (Rose & Richter 2005). For that reason in patients with T1DM exogenous insulin must be injected by intensified insulin therapy or insulin pump therapy. This exogenous insulin docks on the insulin receptor, the insulin receptor substrate (IRS) reacts with G-proteins (guanine nucleotide-binding proteins) on the phosphatidylinositide-3 kinase (PI-3 kinase) and activates GLUT. Furthermore without insulin there is a deficient carbohydrate metabolism while glycogen synthesis, glycogenolysis, gluconeogenesis and glycolysis (Mutschler et al. 2007).

Dependent on dysfunctional insulin release there is also failure in counter regulatory glucagon reaction (Cryer 2012; Ramnanan et al. 2011; Taborsky 2010). In T1DM is no decrease of insulin release while hypoglycemia and thus no increase of α-cell glucagon secretion. So in T1DM there are less endogenous opportunities to avoid hypoglycemia.

1.2. Physiology of type 1 diabetes mellitus in sports

It`s allready known that in T1DM and non type 1 diabetes mellitus (nT1DM) during and after physical activity the glucose utilization increases (Seaquist et al. 2013; Hansen et al. 2013; Reichkendler et al. 2013). During exercise glucose degradation rate in blood plasma increases by enhanced translocating GLUT-4 through skeletal muscles contractions caused by an increase of calcium (Ca^{2+}), adenosine triphosphate (ATP) turnover, adenosinmonophosphate kinases (AMPK), TBC1D1/4, endothelial nitric oxide synthase

(eNOS), p38 mitogen-activated protein kinases (p38 MAPK) and sucrose nonfermenting AMPK-related kinase (SNARK) (Jensen & Richter 2012).

In place of endogenous insulin in nT1DM and exogenous insulin in T1DM muscle contractions activate the GLUT-4 during exercise and as a result glucose consumption increases. In contrast to T1DM patients in healthy subjects endogenous insulin secretion decreases during exercise according to mode, intensity and duration. Therefore in T1DM is a twofold GLUT-4 increase and a high risk of hypoglycemia during exercise.

Furthermore the risk of hypoglycemia exists also post exercise. Several studies have shown that one bout of exercise increases insulin sensitivity for hours which often causes difficulties in insulin regimen (Campbell et al. 2013; Taplin et al. 2010; The Diabetes Research in Children Network (direcnet) Study Group 2006).

1.3. Adaptation of carbohydrate intake and insulin injection before an exercise in type 1 diabetes mellitus

Based on these physiological aspects guidelines describe how to reduce the risk of hypoglycemia during and after exercise (Younk et al. 2011; Hernandez et al. 2000). However, these recommendations are usually very general and unspecific. Several studies have shown, that the regular insulin dose before an exercise leads to hypoglycemia due to the above mentioned mechanisms (American Diabetes Association 2013; Brugnara et al. 2013; Yardly et al. 2013; Dube et al. 2012; Van Bon et al. 2011; Mauras et al. 2010; The Diabetes Research in Children Network (direcnet) Study Group 2006; American Diabetes Association 2004; Sandoval 2004; Biankin 2003). Therefore patients with T1DM have to increase carbohydrate intake and/or to reduce insulin injection dose before and after performing exercise (Younk et al. 2013). The mode of exercise is an influencing factor, which impacts blood glucose levels during and especially after exercise. Some studies demonstrated, that high intensity intermittent exercises lead to a lower decrease in blood glucose level than constant load exercise (Adams 2013; Dube et al. 2012; Harmer et al. 2008; Harmer et al. 2007, Bussau et al. 2006; Guelfi 2005). However, there is a lack in the guidelines to which amount to reduce insulin or increase carbohydrate intake related to different exercise intensities and modes.

2. Pilot study

Objective: To perform sports and exercise in patients with type I diabetes they need to reduce insulin dose or increase carbohydrate intake to avoid hypoglycemia. There is a lack in the guidelines how to reduce insulin or increase carbohydrate intake related to different exercise intensities and modes. The purpose of the present study was to investigate the relationship between the blood glucose concentration and the intensity and the duration of the work load applying a standardized pre exercise glucose and insulin regimen. As a hypothesis we expect an energy expenditure and intensity dependent decrease of blood glucose concentration which offers the opportunity to calculate a critical time (t_{crit}).

Research Design and Methods: One trained male subject with type I diabetes (age: 25 years; weight: 72 kg; high: 1.8 m; VO_2max: 55.4 $ml.kg^{-1}.min^{-1}$; insulin: NovoRapid/Levemir; C-peptide positive, HbA_{1c} 48 $mmol.mol^{-1}$) performed 4 hours after the last insulin/carbohydrate supplement, with a reduction of short time insulin to 40%, a maximal incremental cycle ergometer exercise test (40 W start; 20 $W.min^{-1}$ increments) to determine the first (LTP_1) and the second lactate turn point (LTP_2) by means of computer based linear regression break point analysis. Three phases of energy supply can be detected and separated by LTP_1 and LTP_2. The LTP_1 is characterized as the first increase in blood lactate concentration above baseline. The LTP_2 is detected as the second abrupt increase in lactate between LTP_1 and P_{max} which defines the highest constant workload to give still a lactate steady state (fig. 1). Four constant load ergometer exercise tests (30 min) were performed at 5% P_{max} below and above LTP_1 and LTP_2 (arrows in fig. 1) with a reduction of short time insulin doses of 25% at 5% P_{max} below and above LTP_1 and 50% at 5% P_{max} below LTP_2 and 75% at 5% P_{max} above LTP_2 according to the literature. Heart rate and gas exchange variables were determined continuously, blood lactate concentration (La) and blood glucose concentration were determined at rest, at the end of every workload step, every 5 min in constant load ergometer exercise as well as during 3 and 6 min of active and passive recovery.

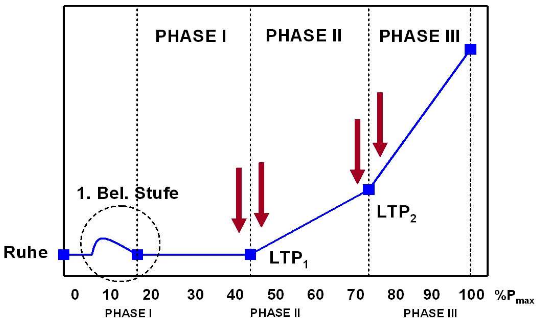

Figure 1: Three phases model of lactate metabolism (LTP_1=Lactate Turn Point 1, LTP_2=Lactate Turn Point 2, La=Lactate, Max=Power maximum; red arrows=Constant load ergometer exercise)

Results: Linear declines of blood glucose were found in all tests. At 5% < LTP_1 glucose decreased from 191 mg/dl to 149 mg/dl, 5% > LTP_1 from 131 md/dl to 96 mg/dl, 5% < LTP_2 from 169 mg/dl to 91 mg/dl and 5% > LTP_2 from 187 mg/dl to 144 mg/dl (early stop because of work load acidosis above LTP_2). The decline of the blood glucose concentrations were calculated by a linear interpolation to t_{crit} (50% rest of the baseline value) without any other supply of carbohydrates (Fig. 2). t_{crit}: 5 % < LTP_1 – 77 min; 5 % > LTP_1 – 31 min; 5% < LTP_2 – 31 min; 5% > LTP_2 – 29 min.

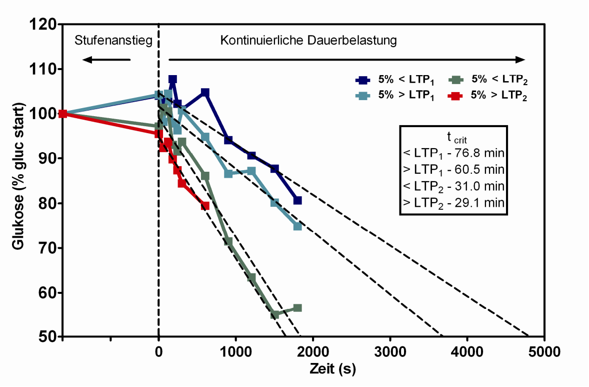

Figure 2: Illustration of the blood glucose levels in relation to the intensity and the duration of the work load

Conclusion: The results show a linear reduction of glucose concentration in relation to the intensity and duration of the work load. We suggest that it is possibly to calculate a critical time for a certain glucose threshold in type I diabetes patients to avoid hypoglycemia during sports and exercise.

3. Study goal and hypothesis

We do know that patients with T1DM must adapt insulin and carbohydrate intake before exercise, but we don`t know the specific individual amount. Therefore we have shown in our pilot study a model how to adapt insulin injection dose (while constant basal insulin use) with respect to standardized intensities and how to calculate a critical time where glucose level in T1DM patients with insulin therapy would fall into hypoglycemia during sports and exercise. Now we have to verify those data with a group of subjects and additional hormonal markers while using basal insulin degludec (Tresiba®, Novo Nordisk).

The aim of the present study is to calculate a critical time by a pre exercise standardized insulin regimen during standardized cycle ergometer exercises dependent on different modes, intensity, duration and energy expenditure as well as counter regulatory hormones. As a hypothesis we expect no hypoglycemia during the cycle ergometer exercises dependent on an standardized pre exercise standardized insulin regimen in relation to the intensity, duration and the energy expenditure of the exercise and no post exercise hypoglycemia.

If the results confirm the hypothesis, this study could be the first recommendation for T1DM patients, how to individually adapt insulin dose before defined exercises without hypoglycemia during and after exercise.

4. Methods

4.1. General study design

This project is a proof of concept study. After the recruitment by the Medical University of Graz (Devision of Endocrinology and Metabolism), at the first screening vistit control of the inclusion and exclusion criteria will be done and subjects will give their signed informed consent. Before testing a 4-week adaptation of basal insulin degludec (Tresiba®, Novo Nordisk) will be done. Each subject will perform a maximal incremental cycle ergometer exercise test to determine exercise markers to prescribe constant load and intermittent exercise tests (Hofmann & Tschakert 2011). Four constant load and three intermittent ergometer exercise tests (30 min) will be performed at 5% P_{max} below and above LTP_1 and LTP_2 with a reduction of short time insulin doses (breakfast-four hours before each test) of 25% at 5% P_{max} below LTP_1 and 50% at 5% P_{max} above LTP_1 and 75% at 5% P_{max} below and above LTP_2 (Tschakert & Hofmann 2013). 24 h before testing subjects will be fitted with a continuous glucose monitoring system (CGMS) for at least 48 h. At 5% P_{max} above LTP_2 there will be no intermittent ergometer exercise test. The same reduction of bolus insulin before testing while be done 15min after testing.

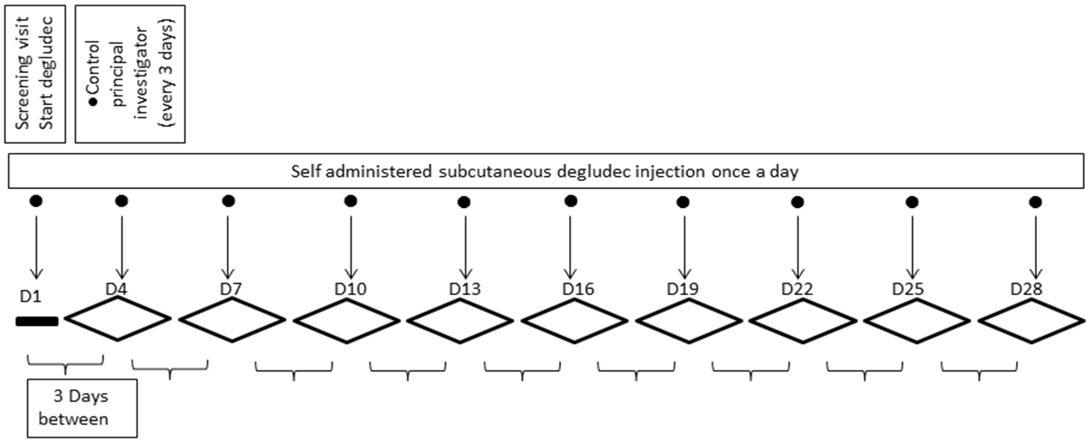

Figure 3: Adaption of insulin degludec

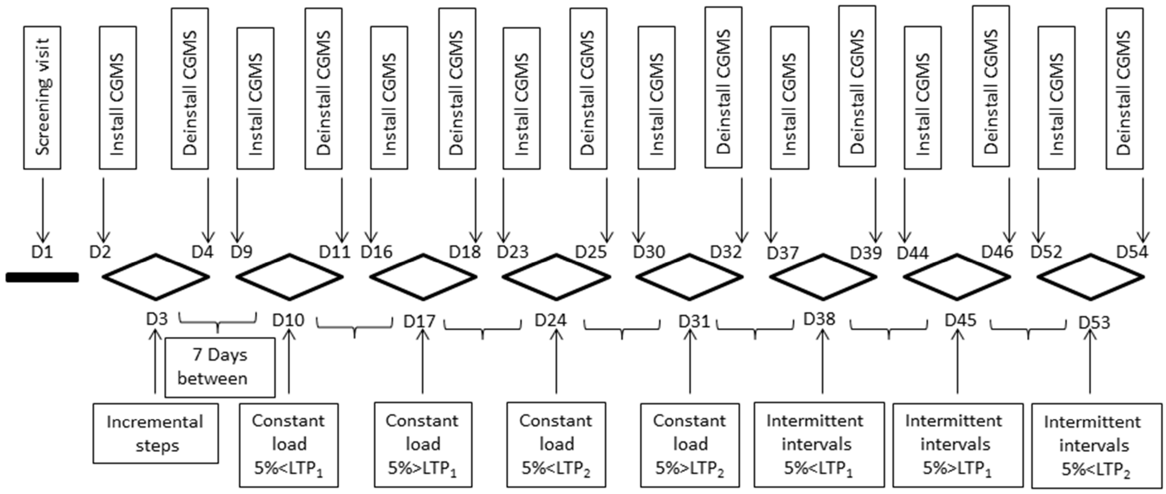

Figure 4: Duration of a study block with one subject. One week between every test to avoid training effect

4.2. Subjects recruitment

Inclusion criteria:

- Subjects must give their signed and dated informed consent before any trial-related activities. Trial-related activities are any procedure that would not have been performed during normal management of the subject
- Male subjects with type 1 diabetes with duration ≥12 month
- Age ≥ 18 to ≤ 35 years, both inclusive
- HbA_{1c} ≤ 64 mmol/mol
- Fasting C-peptide negative (≤ 0.3 nmol/l)
- Treatment with intensified insulin therapy or insulin pump therapy
- No diabetic long term complications
- No other physical and/or mental disease

Exclusion criteria:

- Previous participation (randomisation) in this trial
- History of any illness or disease that, in the opinion of the Investigator might confound the results of the trial
- Use of drugs, which may interfere with the interpretation of trial results or are known to cause clinically relevant interference with insulin action, glucose utilisation, or recovery from hypoglycemia
- Current addiction to alcohol or substances of abuse as determined by the investigator
- Known or suspected allergy to trial products or related products
- Any condition that the investigator feels would interfere with the trial participation or evaluation of data

Testing day inclusion criteria:

- 48h before testing no hypoglycemia
- 24h before testing no alcohol

Testing day exclusion criteria:

- Illness on and/or before testing day
- Low glucose level immediately before testing (< 80 mg/dl)
- Defect CGMS
- Incorrect time of bolus insulin injection (4 hours before testing)
- Mental incapacity, unwillingness, or language barriers precluding adequate understanding or co-operation
- Incorrect amount of bolus insulin injection (last before testing)

4.3. Screening visit

Subjects will receive a subject's number in ascending order. The following will be assessed and recorded in the case report form (CRF):

- Subjects get informed of study design and will give informed consent
- Assessment of inclusion and exclusion criteria
- Demography
- Abuse of drugs, alcohol and smoking habits
- Diagnosis of diabetes
- Body measurements: height (m), weight (kg) and body mass index (BMI)
- Determination of TDD through diabetes diary (Walsh et al. 2011).

4.4. Adaptation of basal insulin degludec (Tresiba®, Novo Nordisk)

Insulin degludec is indicated to be administered once-daily subcutaneous at the same time every day (European Medicines Agency 2013). In T1DM insulin degludec have to be combined with bolus insulin to cover mealtime requirements, so there will be no adaptation in bolus insulin amount. The dosage of insulin degludec will be adjusted every three days individually, with a starting dosage of 70% of the TDD of insulin (European Medicines Agency 2013).

4.5. Calculation of carbohydrate factor and correction factor

To determine the amount of carbohydrate exchanges, which increases blood glucose level, we will calculate the carbohydrate factor (CarbF) and the glucose correction factors (CorrF) according to Walsh et al. (2011):

**CarbF= 5,7*weight (kg)/TDD (U)**
**CorrF= 1960 (mg/dl)/TDD (U)**

The carbohydrate factor indicates how many grams of carbohydrates 1 unit of insulin covers and the correction factor measures how far glucose concentration will fall per 1 unit of insulin (Walsh et al. 2011).

4.6. Install of CGMS and edition carbohydrate supplement

Subjects will be fitted with the CGMS 24h before exercise testing. A hypoglycemic alarm function will be set at 80 mg/dl to avoid hypoglycemia below baseline (70 mg/dl). Standard glucose measurements will be done in a usual way to calibrate the CGMS. Also carbohydrate supplement (Fortimel complete®, Nutricia) will be handed out for the standardized breakfast with the individual amount calculated for each test.

4.7. Maximal incremental cycle ergometer exercise test

Four hours after the last insulin/carbohydrate supplement (Fortimel complete®, Nutricia), with a reduction of short time insulin to 40% and constant basal insulin (calculated amount of carbohydrate intake and dose of insulin injection of the CarbF and CorrF), subjects will perform a maximal incremental cycle ergometer exercise test (40 W start; 20 $W.min^{-1}$ increments) to determine the first (LTP_1) and the second lactate turn point (LTP_2) (Hofmann & Tschakert 2011). Three phases of energy supply can be detected and separated by LTP_1 and LTP_2. The LTP_1 is characterized as the first increase in blood lactate concentration above baseline. The LTP_2 is detected as the second abrupt increase in lactate between LTP_1 and P_{max} which defines the highest constant workload to give still a lactate steady state (fig. 4). Heart rate and gas exchange variables will be determined continuously, blood lactate concentration and blood glucose concentration will be determined at rest at the end of every workload step, every 5 min in constant load ergometer exercise as well as during 3 and 6 min of active and passive recovery. We decided to choose intensities for the constant load tests and the high intensity intermittent exercise tests related to defined phases of energy supply representing common activities. 5% < LTP_1 is like daily activity at low intensity. 5% > LTP_1 is like fast walking at low to moderate intensity. 5% < LTP_2 is equivalent to moderate to high intensity activity or sports below the maximal lactate steady state. 5% > LTP_2 is a high

intensity activity with no more lactate steady state. To simulate sports games we decided to do those high intensity intermittent exercise tests, which are very similar to this type of physical activity.

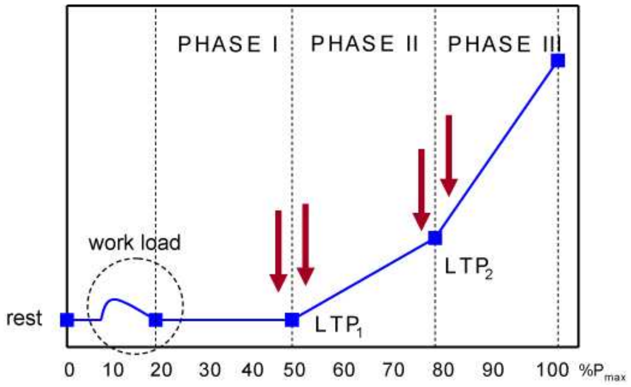

Figure 5: Three phase's model of lactate metabolism (LTP_1=Lactate Turn Point 1, LTP_2=Lactate Turn Point 2, La=Lactate, Max=Power maximum; red arrows=Constant load ergometer exercise and except 5%>LTP_2 for high intensity intermittent exercise)

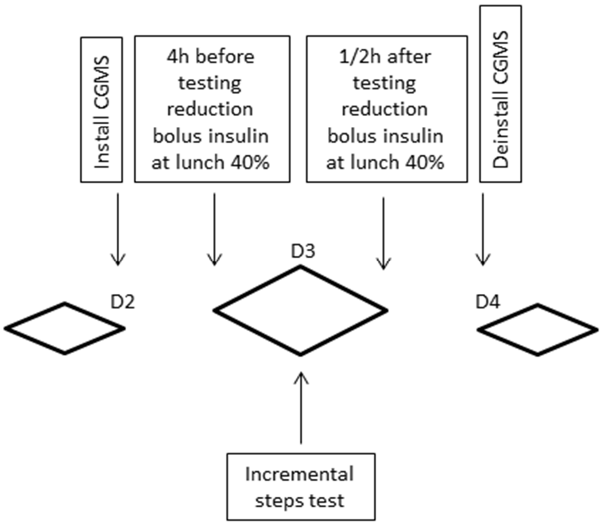

Figure 6: Timetable of incremental steps test

4.8. Constant load test

In all constant load tests heart rate and gas exchange variables will be determined continuously, blood lactate concentration and blood glucose concentration will be determined at rest, at the end of every workload step, every 5 min in constant load ergometer exercise as well as during 3 and 6 min of active and passive recovery. Gas exchange variables will be processed to calculate the energy expenditure and the distribution of glucose and fat metabolism (glucose and fat g/min) (Moser et al. 2013). Furthermore, at the start, in the middle and at the end of exercise venous will be drawn to determine adrenaline, noradrenaline, cortisol, glucagon and somatropin (Bao et al. 2009; Harmer et al. 2007; Bussau et al. 2006; Guelfi et al. 2005; Sandoval et al. 2004). Every 10 minutes blood glucose will be measured additionally from fingertip blood samples to determine the actual blood glucose level to provide hypoglycemia during the exercises and to calibrate CGMS. After the tests glucose concentration will be measured autonomously four times every full hour to avoid post exercise hypoglycemia also by fingertip measures.

The constant load ergometer exercises tests will be performed for 50 min at 5% P_{max} below and above LTP_1 and for 30min at 5% P_{max} below and above LTP_2 (arrows in fig. 5) with a standardized reduction of short time insulin and constant basal insulin (calculated amount of

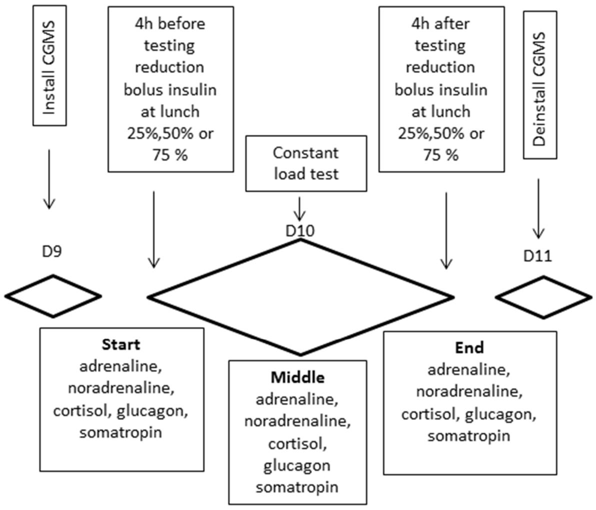

Figure 7: Time table constant load tests

carbohydrate intake and dose of insulin injection of the CarbF and CorrF).

4.9. High intensity intermittent exercise tests

In all high intensity intermittent exercise tests heart rate and gas exchange variables will be determined continuously, blood lactate concentration and blood glucose concentration will be determined at rest, at the end of every workload step, every 5 min in constant load ergometer exercise as well as during 3 and 6 min of active and passive recovery. Gas exchange variables will be processed to calculate the energy expenditure and the distribution of glucose and fat metabolism (glucose and fat g/min) (Moser et al. 2013). Furthermore at the start, in the middle and at the end of exercise adrenaline, noradrenaline, cortisol, glucagon and somatropin will be determined from venous blood samples (Bao et al. 2009; Harmer et al. 2007; Bussau et al. 2006; Guelfi et al. 2005; Sandoval et al. 2004). Every 10 minutes, blood glucose will be measured additionally from fingertip blood samples to determine the actual blood glucose level to provide hypoglycemia during the exercises and to calibrate CGMS. After the tests glucose concentration will be measured autonomously four times every full hour to avoid post exercise hypoglycemia.

The high intermittent exercise tests will be performed for 50 min at 5% P_{max} below and above LTP_1 and for 30min at 5% P_{max} below LTP_2 (arrows in fig. 4) with a standardized reduction of short time insulin and constant basal insulin (calculated amount of carbohydrate intake and dose of insulin injection of the CarbF and CorrF). The high intermittent exercise tests will be performed with the same mean work load (P_{mean}) like in the constant load exercise tests which can be calculated by the following equation:

$$P_{mean} = (P_{peak} * t_{peak} + P_{rec} * t_{rec}) / (t_{peak} + t_{rec})$$

Intervals will be set at P_{max} from 20 sec interspersed by active recovery at 80 % P_{LTP1} whereas the recovery time will be calculated according to the formula by Tschakert & Hofmann (2013).

Table 1: Representation of the whole ergometer testing batch

Intensity	Duration	Mode
1) Maximal incremental cycle ergometer exercise test	All out	All out
2) 5% < LTP_1	30min	Constant load exercise
3) 5% > LTP_1	30min	Constant load exercise
4) 5% < LTP_2	30min	Constant load exercise
5) 5% > LTP_2	30min	Constant load exercise
6) 5% < LTP_1	30min	Intermittent interval
7) 5% > LTP_1	30min	Intermittent interval
8) 5% < LTP_2	30min	Intermittent interval

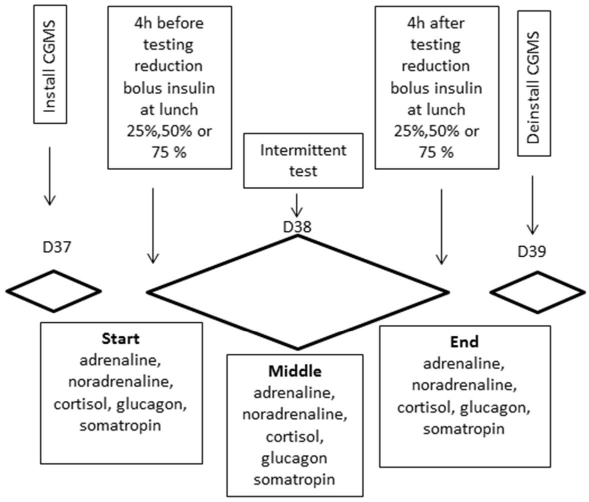

Figure 8: Timetable of high intensity intermittent exercise tests

4.10. Measurements

4.10.1. Anthropometric data and TDD

At the screening visit we will determine body measurements: height (m), weight (kg) and body mass index (BMI) and TDD (U).

4.10.2. Blood glucose, gas exchange, heart rate variables and lactate

In all tests blood glucose and lactate will be measured form blood samples from the earlobe and heart rate and gas exchange variables will be determined continuously. Blood lactate concentration and blood glucose concentration will be determined at rest, at the end of every workload step, every 5 min in constant load ergometer exercise as well as during 3 and 6 min of active and passive recovery. Lactate and glucose concentration will be determined by system Biosen S-line (EKF-Diagnostik, GER). Heart rate will be determined through a 12-lead ECG (ZAN, AUT) and also by Polar PE4000 (Polar Electro, FIN). Gas exchange variables will be determined by an open spiro-ergometry-system (ZAN, AUT). LTP_1 and LTP_2 will be assessed by means of computer based linear regression break point analysis ProSport (Schlemmer, AUT) (Hofmann et al. 2001). Additional fingertip glucose measures will be performed by the subjects own glucose measurement device.

4.10.3. Continuous glucose montoring system (CGMS)

CGMS sensor Dexcom G4 (Dexcom, USA) will be inserted into subcutaneous tissue (postural-lateral abdominal region) 24h prior to each test for 48h, to get a permanent glucose recording pre, during and post exercise and to minimize the risk of hypoglycemia. Blood glucose readings will be stored in the memory of the monitor, which is connected to the sensor. Glucose data will be processed computer based through Dexcom Studio (Dexcom, USA).

4.10.4. Hormones

Adrenaline, noradrenaline, cortisol, glucagon and somatropin will be determined from venous blood samples obtained from a cubital vene at the start, in the middle and at the end of each exercise test. Adrenaline and noradrenaline will be quantified by RIA (DRG Diagnostics, USA) and glucagon by RIA (ICN, USA). Somatropin will be measured using CLIA (Immolite,

Siemens Healthcare Diagnostics, USA) and cortisol by CLIA (CENTAUR Siemens Healthcare Diagnostics, USA).

5. Statistics

Statistical power analysis was done with G*Power 3.1.7 (Faul et al. 2007). A repeated-measures ANOVA design (number of repetitions = 4) was calculated a priori with a medium effect size of 0.5 and an alpha-error of 0.05 based on pilot data. Correlations between repetitions were assumed to equal 0.5. With a sample size of 6 patients the achieved power (beta-1) is greater than 0.95 and is therefore appropriate for a high risk study. In case of dropout we decided to do our study with 7 subjects.

H_0: $\beta_1 = \beta_2 = \beta_3 = \beta_4$ (β……regression coefficient)

H_A: $\beta_1 \neq \beta_2 \neq \beta_3 \neq \beta_4$

Results will be assessed by analysis of variance (ANOVA) for repeated measures with the paired or unpaired t test, Wilcoxon`s rank.-sum test for paired data, or Friedman`s repeated measures by ANOVA on ranks when applicable (GraphPad Prism® Software version 4.0, USA). Values will be presented as mean ± SEM.

6. Time table for subjects

The whole study will last for each subject to 3 months (4 weeks adaption of insulin degludec, 8 weeks of exercise testing – one week between each test). We intend to start the study at 07.01.2014.

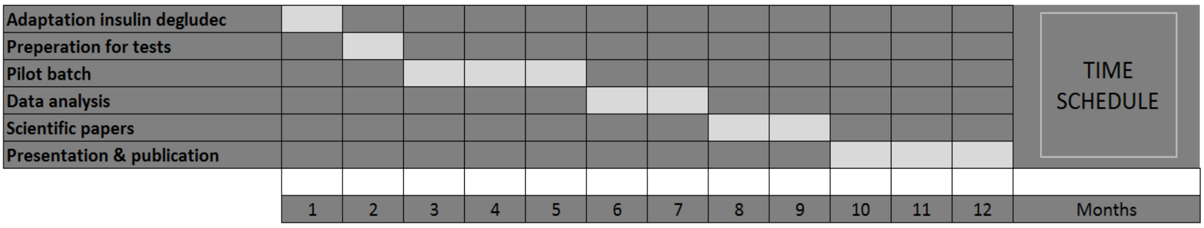

7. Dissemination strategies and potential impact

The protocol information for this study will be registered at an external web site - www.clinicaltrials.gov. Defined goal is to publish our study in prestigious diabetic and sports medical journals and to present the results at scientific conferences. The practice-oriented outcome will also be published as a recommendation to give T1DM patients the possibility to do spontaneous exercise and sports with minimized risk of hypoglycemia.

The results of our project may define data based guidelines how T1DM patients should adapt insulin regimen without risk of hypoglycemia. Worldwide there are 20 million people with T1DM and two-thirds of new diagnoses are younger than 20 years (Patterson et al. 2009; Svensson et al. 2009; Group 2006 & 2007). So, therefore T1DM is a public health problem which influences mostly the younger population. Our strategy will increase the quality of life of patients with T1DM and will minimize the risk of exercise induced hypoglycemia (Davey et al. 2013).

8. Cooperation partners and resources

Our study will be conducted by the Exercise Physiology & Training Research Group, Institute of Sports Science, Univerisity of Graz in cooperation with the Devision of Endocrinolgy and Metabolism of the Medical Univerity of Graz.

All cycle ergometer tests will be performed at the Institute of Sports Science including analysis of lactate, blood glucose, heart rate and gas exchange variables. Recruiment of the subjects before all tests and analyzis of all drawn intravenous blood samples as well as CGMS analysis will be performed by our partners at the Medical University of Graz .

The Exercise Physiology & Training Research Group has a longstanding expertise for the prescription of exercise intensities for scientific studies and clinical studies with respect to sports and exercise. Therefore, the data based adaptation of insulin dose and carbohydrate supplement dependent on exercise intensity seems to be an optimal strategy to verify recommendations for T1DM patients performing sports and intense physical activity. The cooperation partner, the Devision of Endocrinolgy and Metabolism has an outstanding experience and knowhow with respect to glucose pharmatokinetics, important to correctly interprete data in a systemic disease like T1DM. That kind of expert group seems to be the optimal way to protect T1DM patients from exercise induced hypoglycemia.

Research team:

Exercise Physiology & Training Research Group, Institute of Sports Science, University of Graz:

- Ao. Prof. Mag. Dr. Peter Hofmann, FASCM

Project leader, he will have the custody with his expertise in exercise physiology in training concepts in metabolic diseases. He will be present at each cycle ergometer test to supervise the correct sequence of determination blood lactate and glucose. He will also helping analyze all lactate and glucose measurments statistically.

- Dr. med. univ. Werner Gröschl

Medical supervisor while testing period, he will draw blood samples for hormone analysis and he will install CGMS.

- Othmar Moser, Mag.

PhD student, he will be present at all cycle ergometer exercise tests including taking blood glucose und lactate samples from earlobe. Also he will analyze and interprete all tests and will write a PhD theses based on those data.

- Alexander Müller

Labortory technical assistant, helping with cycle ergometer exercise tests, spiroergometry calibration and measures as well as laboratory organization.

<u>Devision of Endocrinology and Metabolism, Medical University of Graz:</u>

- OA. Dr.med. univ. Gerd Köhler

Medical supervisor for endocrinology and recruitment of subjects. He will be the princiapal investigator for adaptaion of insuli degledec. He will be present in the first week at the cycle ergometer exercise test, helping and controlling taking of blood samples.

- Ass.-Prof.[in] Priv.-Doz.[in] Dr.[in] med. univ. Gerlies Treiber

Medical supervisor for endocrinology.

- Univ.-Prof. Dr. med. univ. Thomas Pieber

Medical supervisor for endocrinology. He is the sponsor for the whole pilot study and was helping in creating the study desgn. Furthermore he is head of all medical concerns including analysis of the study and helping writing papers.

9. References

1 Adams P. The impact of brief high-intensity exercise on blood glucose levels. Diabetes, Metabolic Syndrome and Obesity: Targets and Therapy 2013; 6: 113-122.

2 American Diabetes Association. The big blue test: effects of 14 minutes of physical activity on blood glucose levels. Diabetes Care 2013; 36: e21.

3 American Diabetes Association. Physical activity/exercise and diabetes. Diabetes Care 2004; 27 (1): 58-62.

4 Bao S, Briscoe JV, Tate DB, Davis SN. Effects of differing antecedent increases of plasma cortisol on counterregulatory responses during subsequent exercise in type 1 diabetes. Diabetes 2009; 58: 2100-2108.

5 Biankin SA, Jenkins AB, Campbell LV, Choi KL, Forrest QG, Chisholm DJ. Target-seeking behavior of plasma glucose with exercise in type 1 diabetes. Diabetes Care 2003; 26 (2): 297-301.

6 Brooks GA, Fahey TD, White TP, Baldwin KM (2005). Exercise physiology: human bioenergetics and its applications. California: Mayfield publishing company.

7 Brugnara L, Vinaixa M, Murillo S, Samino S, Rodriguez MA, Beltran A, Lerin C, Davison G, Correig X, Novialis A. PloS ONE 2012; 7 (7): 1-8.

8 Bussau VA, Ferreira LD, Jones TW, Fournier PA. The 10-s maximal Sprint: a novel approach to counter an exercise-mediated fall in glycemia in individuals with type 1 diabetes. Diabetes Care 2006; 29 (3): 601-606.

9 Campbell MD, Walker M, Trenell MI, Jakovljevic DG, Stevenson EJ, Bracken RM, Bain SC, West DJ. Large pre- and postexercise rapid-acting insulin reductions preserves glycemia and prevents early- but not late-onset hypoglycemia in patients with type 1 diabetes. Diabetes Care publish ahead of print, published online march 2013: 1-8.

10 Cryer PE. Minireview: glucagon in the pathogenesis of hypoglycemia and hyperglycemia in Diabetes. Endocrinology 2012; 135: 1039-1048.

11 Danaei G, Finucane MM, Lu Y, Singh GM, Cowan MJ, Paciorek CJ, Lin JK, Farzadfar F, Khang Y, Stevens GA, Rao M, Ali MK, Riley LM, Robinson CA, Ezzati M, on behalf of the Global Burden of Metabolic Risk Factors of Chronic Diseases Collaborating Group (Blood Glucose) †. National, regional, and global trends in fasting plasma glucose and diabetes prevalence since 1980: systematic analysis of health examination surveys and epidemiological studies with 370 country-years and 2·7 million participants. The Lancet 2011; 378: 31-40.

12 Davey RJ, Howe W, Paramalingam N, Ferreira LD, Davis EA, Fournier PA, Jones TW. The effect of midday moderate-intensity exercise on post exercise hypoglycemia risk in individuals with type 1 diabetes. J Clin Endocrinol Metab 2013; 1169.

13 Dube MC, Lavaoie C, Weisnagel SJ. Glucose or intermittent high-intensity exercise in glargine/glulisine users with T1DM. Med Sci Sport Exer 2012; doi: 10.1249/MSS.0b013e31826c6ad3.

14 Dube MC, Lavaoie C, Weisnagel SJ. Nutritional strategies to prevent hypoglycemia at exercise in diabetec adolescents. Med Sci Sport Exer 2012; doi: 10.1249/MSS.0b013e3182500a35.

15 European Medicines Agency. Tresiba (insulin degludec): EU summary of pruduct characteristics. 2013. (http://www.emea.europa.eu/ema/. Accessed 01.02.2013

16 Guelfi KJ, Jones TW, Fournier PA. Intermittent high-intensity exercise does not increase the risk of early postexercise hypoglycemia in individuals with type 1 diabetes. Diabetes Care 2005; 28 (2): 416-418.

17 Group S.f.D.i.Y.S. The burden of diabetes mellitus among US youth: prevalence estimates from the SEARCH for diabetes in youth study. Pediatrics 2006; 118: 1510-1518.

18 Group T.W.G.f.t.S.f.D.i.Y.S. Incidence of diabetes in youth in the United States. JAMA, J Am Med Assoc 2007; 297: 2716-2724.

19 Han S, Donelan W, Wang H, Reeves W, Yang L-J. Novel autoantigens in type 1 diabetes. Am J Transl Re 2013; 5 (4): 379-392.

20 Hansen AL, Cartensen B, Helge JW, Johansen NB, Gram B, Christiansen JS, Brage S, Lauritzen T, Jørgensen ME, Aadahl M, Witte DR, ADDITION-Denmark Steering Committee. Combined heart rate- and accelerometer-assessed physical activity energy expenditure and associations with glucose homeostasis markers in a population at high risk of developing diabetes: The ADDITION-PRO Study. Diabetes Care 2013; June 11: doi: 10.2337/dc12-2671.

21 Harmer AR, Chisholm DJ, McKenna MJ, Hunter SK, Ruell PA, Naylor JM, Maxwell LJ, Flack JR. Sprint training increases muscle oxidative metabolism during high-intensity exercise in patients with type 1 diabetes. Diabetes Care 2008; 31 (11): 2097-2102.

22 Harmer AR, Chisholm DJ, McKenna MJ, Morris NR, Thom JM, Bennett G, Flack JR. High-intensity training improves plasma glucose and acid-base regulation during intermittent maximal exercise in type 1 diabetes. Diabetes Care 2007; 30 (5): 1269-1271.

23 Hofmann P, Tschakert G. Special needs to prescribe exercise intensity for scientific studies. Cardiol Res Pract 2011; Article ID 209302, 10 pages, doi:10.4061/2011/209302.

24 Hofmann P, Von Duvillard SP, Seibert FJ, Pokan R, Wonisch M, Lemura LM, Schwaberger G. %HRmax target heart rate is dependent on heart rate performance curve deflection. Med Sci Sports Exerc 2001; 33(10):1726-1731.

25 Jensen TE, Richter EA. Regulation of glucose and glycogen metabolism during and after exercise. J Phiol 2012; 590.5: 1069-1076.

26 Larsson HE, Jönsson I, Lernmark Ǎ, Ivarsson S, Radtke JR, Hampe CS, DiPiS, Type 1 Diabetes Trial Network. Decline in titers of anti-idiotypic antibodies specific to autoantibodies to GAD65 (GAD65Ab) precedes development of GAD65Ab and type 1 diabetes. PLoS ONE. 2013; 8 (6): e65173.

27 Lind M, Oden A, Fahlen M, Eliasson B. The true value of HbA1c as a predictor of diabetec compications: simulations of HbA1c variables. PloS ONE. 2009; 4 (2): 1-6.

28 Long AE, Gillespie KM, Rokni S, Bingley PJ, Williams AJK. Rising incidence of type 1 diabetes is associated with altered immunophenotype at diagnosis. Diabetes 2012; 61: 683-686.

29 Mauras N, Xing D, Fox LA, Englert K, Darmaun D. Effects of glutamine on glycemic control during and after exercise in adolescents with type 1 diabetes. Diabetes Care 2010; 33 (9): 1951-1953.

30 Moser O, Müller A, Gröschl W, Köhler G, Hofmann P. Zusammenhang zwischen dem Abfall der Blutzucker-Konzentration und definierten physischen Belastungen am Fahrrad-Ergometer bei Diabetes mellitus Typ I - Einzelfallstudie. Abstract-CD Diabetes Kongress 2013 / 48. Jahrestagung der Deutschen Diabetes Gesellschaft 2013; P292: ISSN 2192-1024 Jahrgang.

31 Mutschler E, Schaible HG, Vaupel P (2007). Anatomie Physiologie Pathophysiologie des Menschen. Stuttgart: Wissenschaftlicher Verlagsgesellschaft mbH.

32 Oyer DS. The science of hypoglycemia in patients with diabetes. Curr Diabetes Rev 2013; 9 (3): 195-208.

33 Patterson CC, Dahlqist GG, Gyürüs E, Green A, Soltész G. Incidence trends for childhood type 1 diabetes in Europe during 1989-2003 and predicted new cases 2005-20: a multicenter prospective registration study. The Lancet 2009; 373: 2027-2033.

34 Ramnanan CJ, Edgerton DS, Kraft G, Cherrington AD. Physiologic action of glucagon on liver glucose metabolism. Diabetes Obes Metab. 2011; 13 (1): 118-125.

35 Reichkendler MH, Auerbach P, Rosenkilde M, Christensen AN, Holm S, Petersen MB, Lagerberg A, Larsson HB, Rostrup E, Mosbech TH, Sjödin A, Kjaer A, Ploug T, Hoejgaard L, Stallknecht BM. Exercise training favors increased insulin-stimulated glucose uptake in skeletal muscle in contrast to adipose tissue: A randomized study using FDG PET imaging. Am J Physiol Endocrinol Metab 2013; 1522-1555.

36 Rose AJ, Richter EA. Skeletal muscle glucose uptake during exercise: how is it regulated? Physiology 2005; 20: 260-270

37 Sandoval DA, Aftab Guy DL, Richardson MA, Ertl AC, Davis SN. Effects of low and moderate antecedent exercise on the counterregulatory responses to subsequent hypoglycemia in type 1 diabetes. Diabetes 2004; 53: 1798-1806.

38 Sluik D, Boeing H, Montonen J, Kaaks R, Lukanova A, Sandbaek A, Overvad K, Arriola L, Ardanaz E, Saieva C, Grioni S, Tumino R, Sacerdote C, Mattiello A, Spijkerman AM, van der A DL, Beulens JWJ, van Dieren S, Nilsson PM, Groop LC, Franks PW, Rolandsson O, Bueno-de-Mesquita B, Nöthlings U. HbA1c measured in stored

erythrocytes is positively linearly associated with mortality in individuals with diabetes mellitus. PloS ONE. 2012; 7 (6): 1-9.

39 Seaquist ER, Anderson J, Childs B, Cryer P, Dagogo-Jack S, Fish L, Heller SR, Rodriguez H, Rosenzweig J, Vigersky R. Hypoglycemia and diabetes: a report of a workgroup of the American Diabetes Association and The Endocrine Society. J Clin Endocrinol Metab 2013; 98: 1845-1859.

40 Svensson J, Lyngaae-Jorgenson A, Carstensen B, Simonsen LB, Mortensen HB. Long-term trends in the incidence of type 1 diabetes in Denmark: the seasonal variation changes over time. Pediatr. Diabetes 2009; 10: 248-254.

41 Taplin CE, Cobry E, Messer L, McFann K, Chase HP, Faillo-Scharer R. Preventing post-exercise nocturnal hypoglycemia in children with type 1 diabetes by suspending basal insulin. J Pediatr 2010; 157 (5): 784-788.

42 The Diabetes Control and Complications Trial/Epidemiology of Diabetes Interventions and Complications (DCCT/EDIC) Study Research Group. Intensive diabetes treatment and cardiovascular disease in patients with Type 1 diabetes. N. Engl. J. Med. 2005; 353 (25): 2643-2653.

43 The Diabetes Research in Children Network (direcnet) Study Group. Prevention of hypoglycemia during exercise in children with type 1 diabetes by suspending basal insulin. Diabetes Care 2006; 29 (10): 2200-2204.

44 Taborsky GJ. The physiology of glucagon. Journal of Diabetes Science and Technology. 2010; 4 (6): 1338-1344.

45 Tschakert G, Hofmann P. High-Intensity intermittent exercise - methodological and physiological aspects. Int J Sport Physiol Perf 2013.

46 Tschakert G, Gröschl W, Schwaberger G, Von Duvillard SP, Hofmann P. Prescription for aerobic high-intensity interval training by means of incremental exercise tests markers. Med Sci Sport Exer 2009; 41(5): 430.

47 Von Bon AC, Verbitskiy E, Von Basum G, Hoekstra JBL, DeVries JH. Exercise in cloosed-loop control: a major hurdle. Journal of Diabetes Science and Technology 2011; 5 (6): 1337-1341.

48 Yardley JE, Kenny GP, Perkins BA, Riddell MC, Balaa N, Malcolm J, Boulay P, Khandwala F, Sigal TJ. Resistance versus aerobic exercise: acute effects on glycemia in type 1 diabetes. Diabetes Care 2013; 36 (3): 537-542.

49 Younk LM, Mikeladze M, Tate D, Davis SN. Exercise-related hypoglycemia in diabetes mellitus. Expert Rev Endocrinol Metab 2011; 6 (1): 93-108.
